# A Quadruplex Real-Time PCR Assay for the Rapid Detection and Differentiation of the Most Relevant Members of the *B*. *pseudomallei* Complex: *B*. *mallei*, *B*. *pseudomallei*, and *B*. *thailandensis*

**DOI:** 10.1371/journal.pone.0164006

**Published:** 2016-10-13

**Authors:** Chinn-Woan Lowe, Benjamin A. Satterfield, Daniel B. Nelson, Joseph D. Thiriot, Michael J. Heder, Jordon K. March, David S. Drake, Cynthia S. Lew, Annette J. Bunnell, Emily S. Moore, Kim L. O'Neill, Richard A. Robison

**Affiliations:** Department of Microbiology and Molecular Biology, Brigham Young University, Provo, UT, 84602, United States of America; Naval Research Laboratory, UNITED STATES

## Abstract

The *Burkholderia pseudomallei* complex classically consisted of *B*. *mallei*, *B*. *pseudomallei*, and *B*. *thailandensis*, but has now expanded to include *B*. *oklahomensis*, *B*. *humptydooensis*, and three unassigned *Burkholderia* clades. Methods for detecting and differentiating the *B*. *pseudomallei* complex has been the topic of recent research due to phenotypic and genotypic similarities of these species. *B*. *mallei* and *B*. *pseudomallei* are recognized as CDC Tier 1 select agents, and are the causative agents of glanders and melioidosis, respectively. Although *B*. *thailandensis* and *B*. *oklahomensis* are generally avirulent, both display similar phenotypic characteristics to that of *B*. *pseudomallei*. *B*. *humptydooensis* and the *Burkholderia* clades are genetically similar to the *B*. *pseudomallei* complex, and are not associated with disease. Optimal identification of these species remains problematic, and PCR-based methods can resolve issues with *B*. *pseudomallei* complex detection and differentiation. Currently, no PCR assay is available that detects the major species of the *B*. *pseudomallei* complex. A real-time PCR assay in a multiplex single-tube format was developed to simultaneously detect and differentiate *B*. *mallei*, *B*. *pseudomallei*, and *B*. *thailandensis*, and a common sequence found in *B*. *pseudomallei*, *B*. *mallei*, *B*. *thailandensis*, and *B*. *oklahomensis*. A total of 309 *Burkholderia* isolates and 5 other bacterial species were evaluated. The assay was 100% sensitive and specific, demonstrated sensitivity beyond culture and GC methods for the isolates tested, and is completed in about an hour with a detection limit between 2.6pg and 48.9pg of gDNA. Bioinformatic analyses also showed the assay is likely 100% specific and sensitive for all 84 fully sequenced *B*. *pseudomallei*, *B*. *mallei*, *B*. *thailandensis*, and *B*. *oklahomensis* strains currently available in GenBank. For these reasons, this assay could be a rapid and sensitive tool in the detection and differentiation for those species of the *B*. *pseudomallei* complex with recognized clinical and practical significance.

## Introduction

Glanders, a disease caused by the Gram-negative bacillus *Burkholderia mallei*, is endemic in Africa, Asia, the Middle East, and Central and South America [1]. This bacterium does not survive well outside of the host and primarily infects equine populations such as horses, donkeys, and mules. Equine infection is largely due to ingestion of feed or water contaminated with nasal discharges from infected animals [1]. Human *B*. *mallei* infection is uncommon, but certain groups are at risk including veterinarians, slaughterhouse workers, equine butchers, equine handlers, and laboratory workers. For humans, infection is usually acquired by contact with infectious material through breaks in the skin or mucous membranes such as the eyes, nose, and mouth. The disease has a 95% case fatality rate for untreated septicemia [2] with death occurring in 7–10 days [3], and a 50% case fatality rate in antibiotic-treated patients [2].

Melioidosis, a disease caused by the saprophytic Gram-negative bacillus *B*. *pseudomallei*, is endemic in the sub-tropical areas of Southeast Asia and northern Australia. Unlike *B*. *mallei*, *B*. *pseudomallei* can easily survive outside of an infected host. The bacterium is commonly found in wet soils and stagnant waters, such as rice paddies, throughout endemic regions. High risk groups involve individuals who are in direct contact with wet soil including rice paddy workers, indigenous groups located in southern and eastern Asia, sub-tropical travelers, and individuals who are afflicted with immunosuppressive illnesses including diabetes mellitus, cirrhosis, thalassemia, renal disease, and alcoholism [4–6]. Melioidosis is acquired through inhalation, contact with cuts/wounds, or occasionally through ingestion of contaminated water [7, 8] with overall mortality rates of 40% in northern Thailand [8], 39% in Singapore [9], and 19% in Australia [5]. The infectious threat posed by melioidosis is gaining wider recognition because of the increasing number of melioidosis cases reported outside of Southeast Asia and northern Australia [10–13].

*B*. *thailandensis*, a saprophytic Gram-negative bacillus, is readily found in moist soils and stagnant water throughout Southeast Asia and northern Australia. In 1988, *B*. *thailandensis* was proposed as a new species distinct from *B*. *pseudomallei* because of differences in 16S rRNA gene (rDNA) sequences, biochemical profiles, and virulence traits [14]. *B*. *oklahomensis*, *B*. *humptydooensis*, and three unassigned *Burkholderia* clades A, B, C (respectively represented as BDU 5, BDU 8, and MSMB 0265) are *B*. *thailandensis-*like strains. The *Burkholderia* clades have not been assigned a species but are most related to *B*. *oklahomensis* [15]. *B*. *oklahomensis* was isolated in soil from Oklahoma, USA, *B*. *humptydooensis* was isolated from bore water from Humpty Doo, Northern Australia, and the *Burkholderia* clades are environmental isolates from Northern Australia [15–18]. The virulence of *B*. *humptydooensis* and the *Burkholderia* clades are currently unknown, although no human cases have been reported. *B*. *thailandensis* and *B*. *oklahomensis* are generally considered avirulent for mammals [19], but rare cases of disease have been documented [20–24]. *B*. *thailandensis* shares several virulence factor homologs with *B*. *mallei* and *B*. *pseudomallei*, making *B*. *thailandensis* a popular model organism to study *Burkholderia* pathogenesis. In addition, *B*. *thailandensis* and *B*. *oklahomensis* displays very similar characteristics to that of *B*. *pseudomallei* by various tests [16, 17, 25]. *B*. *thailandensis* and *B*. *humptydooensis* are known to co-localize with *B*. *pseudomallei* in the environment [16]. Therefore, these species can easily contaminate samples and/or produce false-positive results in current assays for melioidosis, leading to unnecessary treatment.

As previously stated, cases of illness from *B*. *thailandensis* and *B*. *thailandensis*-like strains have been reported, but may not fully represent the number of true cases of illness, due to limitations in current detection methods for these species. *B*. *mallei* and *B*. *pseudomallei* require BSL-3 management due to their virulence and classification by the CDC as Tier 1 select agents, and both pathogens have a history of bioweapon use [7, 26–30]. *B*. *mallei* and *B*. *pseudomallei* are known to be phylogenetically similar and it is generally recognized that *B*. *mallei* evolved as a strict pathogen from *B*. *pseudomallei* [31, 32]. *B*. *thailandensis*, *B*. *oklahomensis*, and *B*. *humptydooensis* also shares several phenotypic and molecular characteristics with *B*. *mallei* and *B*. *pseudomallei* [16–18, 33–37]. The *B*. *pseudomallei* complex was once comprised of *B*. *pseudomallei*, *B*. *mallei*, and *B*. *thailandensis*, but was recently expanded to include *B*. *oklahomensis*, *B*. *humptydooensis*, and three unassigned *Burkholderia* clades A, B, and C [15], due to the similarities of these species to those previously described.

Several methods have been developed for detection of various combinations of these species, which include serologic tests [38–42], commercial biochemical tests [43–46], Gas Chromatography Fatty Acid Methyl Esters (GC-FAME) [47], Gas Liquid Chromatography-Fatty Acid Methyl Esters [48], microscopic methods [49–51], Matrix Assisted Laser Desorption/Ionization Time-of-Flight [52], PCR subtractive hybridization technique [53], PCR-Restriction Fragment Length Polymorphisms [54] and gene sequencing [15, 36, 55, 56]. Serologic tests may be unreliable in endemic areas due to high seroconversion rates [7] for those previously exposed to the organism. Therefore, these serologic tests have low sensitivity and specificity in areas of endemicity [38, 57], but may prove useful in non-endemic areas. Biochemical assays have misidentified *B*. *pseudomallei* as *Pseudomonas spp*, *B*. *vietnamiensis*, *Stenotrophomonas maltophilia*, and *Chromobacterium violaceum* [43, 44, 46]. However, many of these methods require culture growth, which may take up to seven days to confirm a diagnosis. Consequently, improperly treated patients may die before receiving a correct diagnosis. Direct testing methods on clinical samples would prove most useful, but due to the low numbers of these organisms in many clinical samples, detection can be difficult.

With the exception of *B*. *humptydooensis* and the *Burkholderia* clades, culture growth is the gold-standard for clinical detection of the *B*. *pseudomallei* complex [21, 23, 58], but the high mortality rates of diseases caused by *B*. *mallei* and *B*. *pseudomallei*, their potential use as bioweapons, their low infectious dose, variable incubation periods, and diagnostic difficulties, necessitates the development of more rapid and accurate detection assays. The development of PCR technologies has revolutionized diagnostic testing methods for many microbial pathogens. These assays have become widely used due to their low detection limit, specificity, and speed. PCR-based assays for the rapid detection and differentiation of the *B*. *pseudomallei* complex have been the topic of much recent research. Optimal identification of these species remains problematic, due to difficulties in developing a sensitive and selective assay [59]. There are several real-time PCR assays that detect a single species in the *B*. *pseudomallei* complex [59–65]. Some of these assays were further validated with additional *Burkholderia* species DNA or evaluated for clinical and environmental use [60, 66–70]. Several PCR assays can detect multiple species of the *B*. *pseudomallei* complex, but due to their genetic similarity, these assays have not included species-specific primers. Current assays that detect and differentiate the *B*. *pseudomallei* complex frequently have only one species-specific primer set paired with other primers that detect the species as a complex. When all primers are tested against a sample, a unique amplification profile is created. Therefore, all primer sets are necessary to detect and differentiate the *B*. *pseudomallei* complex. These non-species specific assays are accomplished by multiplex PCR by Koh *et al*. [71] and Lee *et al*. [72] and real-time PCR by Thibault *et al*. [25]. Although these PCR studies have shown discrimination, they employ traditional PCR methods which have inherent limitations on throughput. The real-time PCR assay by Thibault *et al*. [25] has the potential for higher throughput, but requires three separate real-time PCR reactions in order to correctly identify *B*. *pseudomallei*, *B*. *mallei*, and *B*. *thailandensis*. Some follow-up studies of Thibault *et al*'s *B*. *mallei* and *B*. *pseudomallei* assays [25] have reported a decreased specificity in the detection of non-*B*. *pseudomallei* complex species [59, 64, 65, 70, 73]. Currently, only three real-time PCR species-specific assays can detect some members of the *B*. *pseudomallei* complex. Two duplex real-time PCR assays detect *B*. *mallei* and *B*. *pseudomallei* based on a single-nucleotide polymorphism (SNP) [74, 75]. The third real-time PCR species-specific assay detects and differentiates *B*. *pseudomallei* from the *B*. *thailandensis*, *B*. *thailandensis*-like, and *B*. *oklahomensis* group [76]. To date, no PCR assay has been developed that can specifically detect and differentiate between *B*. *mallei*, *B*. *pseudomallei*, and *B*. *thailandensis* in a single-tube format. Therefore, the purpose of this study was to develop and validate a real-time PCR assay that could quickly, accurately, and precisely detect and differentiate several species of the *B*. *pseudomallei* complex. This has potential applications in rapidly determining sources of infection in human patients in endemic regions, particularly in detecting contaminated environments like soil, and in differentiating between pathogenic and non-pathogenic members of the *B*. *pseudomallei* complex.

## Materials and Methods

### Bacterial Isolates and Culture Conditions

The 314 bacterial isolates used in this study (S1 Table) were acquired from the American Type Culture Collection (ATCC), Culture Collection, University of Göteborg (CCUG), Centers for Disease Control and Prevention (CDC), National Collection of Type Cultures (NCTC), Public Health England (PHE), Royal Darwin Hospital (RDH), University of Calgary (UC), and Utah Department of Health (UDH). The isolates were grown on Columbia agar (Remel) at 28–37°C for 1–3 days prior to DNA extraction. A genetically diverse panel of isolates was used in this study, which consist of human, animal, and environmental samples originally isolated from 24 countries (S2 Table).

### DNA Extraction

Total genomic DNA was extracted from each isolate by first suspending cells grown on Columbia agar plates in 510 μL of Tris/EDTA buffer [10 mM Tris/HCl (pH 8.0), 1 mM EDTA] containing 1.8 μg μL^-1^ lysozyme, and incubating for 40 minutes at 37°C. To this tube, 540 μL of bacterial lysis buffer and 100 μL of proteinase K were added, after which it was incubated for 10 min at 65°C followed by an automated DNA extraction performed with a Roche MagNA Pure LC system (Roche Diagnostics), using the Roche MagNA Pure LC DNA Isolation Kit III as recommended by the manufacturer. DNAs were tested for biological growth by plating 10% of the DNA sample volume on Columbia Agar (Remel) at 28–37°C for five days. When the DNA samples passed sterility, DNA concentrations were measured with a TBS-380 Fluorometer (Promega) using the Quant-iT PicoGreen dsDNA assay kit P11496 (Invitrogen).

### Primer and Probe Design

DNA sequences unique to *B*. *mallei*, *B*. *pseudomallei*, and *B*. *thailandensis*, as well as a unique target common among *B*. *pseudomallei*, *B*. *mallei*, and *B*. *thailandensis* were obtained from NCBI GenBank (http://www.ncbi.nlm.nih.gov/genbank). Several genes were targeted for *B*. *pseudomallei* complex detection and differentiation. A gene that encodes a 16.5 kDa hypothetical protein (Accession number YP_001024199 Locus tag = BMA10229_0375) was reported to be unique to *B*. *mallei* [77]. *Orf11* (Accession number AF074878) was reported to be unique to *B*. *pseudomallei* [25, 78]. A gene that encodes a 70 kDa hypothetical protein (Accession CP000086 Locus tag = BTH_I1515) was reported to be specific to *B*. *thailandensis* [79]. Flagellar structural protein, *fliC* (Accession numbers U82287, AF084815, AF081500) was reported to be exclusive to the entire *B*. *pseudomallei* complex [80, 81]. These regions were used to design the primers and probes reported in this study. All primers and 5'-hydrolysis dual-labeled probes (Table 1), except for the *fliC* dual-labeled probe, were designed using the *PrimerQuest* algorithms from Integrated DNA Technologies (IDT) (http://www.idtdna.com/primerquest/Home/Index). The *fliC* dual-labeled probe was designed by comparing *fliC* gene sequences of *B*. *pseudomallei*, *B*. *mallei*, *B*. *thailandensis*, and *B*. *vandii* ATCC 51545 (GenBank Accession KM242678). All oligo sequences were selected for proper GC content, optimal annealing temperatures, and lack of hairpin structures. A thorough NCBI BLASTn search and/or analysis of sequence alignments using MEGA 6.0 [82] were performed to ensure both primer and probe specificity and lack of homology with sequences from other organisms. Oligos were considered for further evaluation if NCBI BLASTn had expect-values close to zero and identity values close to 100% for the intended species. Probes were fluorescently labeled as follows: 16.5 kDa (*B*. *mallei*) with Cy5, *orf11* (*B*. *pseudomallei*) with FAM, 70 kDa (*B*. *thailandensis*) with Tex615, and *fliC* (*B*. *pseudomallei*, *B*. *mallei*, and *B*. *thailandensis* group) with Cy3.

**Table 1:** Primer and probe sequences of the quadruplex assay.

Target gene (species)	Oligo	Sequence (5’ → 3’)	Amplicon Size (bp)
**16.5 kDa**			
*B*. *mallei*	Forward	CGT TCG AGC TCA GCA ACC TCG TTA	85
	Reverse	AAG CGG TGA TGG ACC GCT GTA T	
	Probe	Cy5 -CAG TAT CCA GGT TTC ACC GCG CTC GAC-IAbRQ	
***Orf11***			
*B*. *pseudomallei*	Forward	AAC ACT GAC AAG TGG CCC TAT GGA	185
	Reverse	TCC GAT CGG TTT CGA ATA ACG GGT	
	Probe	FAM -ACG ATC TCC-ZEN-GAG AAC GCA CTG AAC A-IAbFQ	
**70 kDa**			
*B*. *thailandensis*	Forward	AAC CTG AGG CAA CGC AAG AAG AAG	99
	Reverse	TTT CTT CAC GCA TTC CCA ACC CTG	
	Probe	Tex615 -TCA AGG CGA GCT GTG CCG ACA ACA A-IAbRQ	
***fliC***			
*Bp*, *Bm*, *& Bt group*	Forward	ACG GTC AAC AAY CTG CAG GCA A	143
	Reverse	TTC GCG GTT TCC TGA GCR AAG TC	
	Probe	Cy3- GGC TCG AAC AAC CTC GCG CAR G-IAbRQ	

IAbRQ, Iowa Black RQ Quencher; IAbFQ, Iowa Black FQ Quencher; ZEN, ZEN Internal Quencher; *Bp*, *B*. *pseudomallei*; *Bm*, *B*. *mallei*, *Bt*, *B*. *thailandensis*

### Sequence Analysis

Sequencing was used to further analyze three *Burkholderia* strains. A portion of the *fliC* gene of *B*. *vandii* ATCC 51545 was amplified with forward primer 5' CGG CTT CAC GTT CAC CGA YCA G and reverse primer 5' GCA GGA GCT TCA GCA CTT GCT G. The 16S rRNA gene of *B*. *pseudomallei* 135 and MSHR 1816 was amplified using in-house 16S rDNA universal forward primer 5' ACT CCT ACG GGA GGC AGC AGT and reverse primer 5' TAC GGT TAC CTT GTT ACG ACT T. For every reaction, a master mix of 25 μL was prepared using 1x Hot Start Mix RTG Master Mix (GE Healthcare) and the following: 500 nM of each amplification primer, 2 μL target DNA, and PCR H_2_O to 25 μL. The mixtures were loaded into 0.2 ml PCR tubes, and PCR was performed using a GeneAmp PCR System 9700 (Applied Biosystems). The reaction mix for the *fliC* product was initially denatured at 95°C for 3 min followed by 30 cycles of 95°C for 15 sec, 61°C for 30 sec, 72°C for 1 min, and a final extension of 72°C for 5 min. The reaction mix for the 16S rDNA product was initially denatured at 95°C for 5 min followed by 30 cycles of 94°C for 15 sec, 58°C for 30 sec, 72°C for 1 min, and a final extension of 72°C for 5 min.

PCR products were purified with the Exo-SAP IT (Affymetrix) using manufacturer's recommendations. The purified PCR product was sequenced using 500 nM of each sequencing primer. The *fliC* forward sequencing primer 5' AAC GCA GCA AGC CAA CGC and reverse primer 5' TCT GGA TTT GCG ATT GAG CCG AC were used. The amplification primers for the 16S rDNA product and forward primer 5' AGA GTT TGA TCC TGG CTC AG were used as sequencing primers. Sequencing was performed with a BigDye Terminator version 3.1 cycle sequencing kit (Life Technologies) as per manufacturer's recommendations. Sequencing products were purified with a Sephadex spin (GE Healthcare) column and resolved with a 3730 DNA Analyzer (Life Technologies). The region of interest was re-amplified and re-sequenced at least twice to ensure sequence accuracy.

MEGA 6.0 was used to align sequences [82], and the sequences were analyzed using the Ribosomal Database Project (http://rdp.cme.msu.edu/seqmatch/seqmatch_intro.jsp), Greengenes (http://greengenes.lbl.gov/cgi-bin/nph-index.cgi), and NCBI BLASTn (http://blast.ncbi.nlm.nih.gov/Blast.cgi) databases.

IDT's DNA Thermodynamics & Hybridization (http://biophysics.idtdna.com/) software was used to predict how SNPs would affect oligo binding efficiency. All components of the *orf11* assay, *fliC* probe, and *fliC* forward primer were further examined using this software. T_m_, Gibbs Energy, Enthalpy, Entropy, and fraction of duplex values were generated for each match or mismatch along with a hybridization mismatch profile.

### Real-time PCR Singleplex Optimization

Important parameter variables such as the number of PCR cycles, cycle temperatures, and length of annealing and elongation steps, were all optimized. Primers were first evaluated with SYBR Green to optimize cycle temperatures and times. For every reaction, a master mix of 25 μL was prepared using 1x Hot Start Mix RTG Master Mix (GE Healthcare) and the following: forward and reverse primers at 500 nM, 2 μL target DNA, 1.25 μL SYBR Green at a 25x concentration and PCR H_2_O to 25 μL. The mixtures were loaded into 25 μL Cepheid PCR tubes, and PCR was performed using a SmartCycler II (Cepheid). During the cycling phase, the annealing/extension temperature was varied from 57°C to 65°C in single degree increments to maximize the reaction. The optimized procedure identified and used for the 16.5 kDa singleplex assay was 550 nM of each primer, 300 nM of probe (Table 1) with an initial denaturation at 95°C for 150 s followed by 40 cycles of 95°C for 15 s, then 61°C for 50 s. The optimized protocol identified and used for the *orf11* singleplex assay was 450 nM of each primer, 300 nM of probe with an initial denaturation at 95°C for 150 s followed by 35 cycles of 95°C for 15 s, then 61°C for 45 s. The 70 kDa assay was optimized using 400 nm of each primer and 250 nm of probe, and the *fliC* assay used 450 nM of each primer and 400 nM of each probe. The optimized procedure identified and used for both the 70 kDa and *fliC* singleplex assays was an initial denaturation at 95°C for 150 s followed by 35 cycles of 95°C for 15 s, then 61°C for 50 s. A sample was determined positive if it crossed a fluorescence threshold of 30 for the 16.5 kDa test before cycle 40 (a C_T_ value of less than 40). A sample was determined positive if it crossed a fluorescence threshold of 30 before cycle 35 for the *orf11* and 70 kDa assay. Lastly, the sample was determined positive if the fluorescence threshold crossed 15 for the *fliC* assay before cycle 35. DNA from near-neighbors and no template were used as negative controls.

### Multiplexing the Four Singleplex Real-time PCR Assays

Once the single reaction conditions were optimized, the four assays were multiplexed (quadruplexed) into a single-tube format. For each reaction, 1.5x Hot Start Mix RTG master mix (GE Healthcare) was added to a mixture of 600 nM of each primer and 400 nM of probe for the 16.5 kDa test, 200nM of each primer and probe for the *orf11* protocol, 250 nM of each primer and 200 nM of each probe for the 70 kDa test, and 400 nM of each primer and 250 nM of probe for the *fliC* assay. Two μl of target DNA and PCR-grade H_2_O were added for a total reaction volume of 25 μL. Thermal cycling conditions were an initial denaturation at 95°C for 150 s followed by 35 cycles of 95°C for 15 s, then 61°C for 50 s. A sample was determined to be positive if the 16.5 kDa, *orf11*, 70 kDa, and *fliC* tests crossed a fluorescence threshold of 15, 20, 30, and 15, respectively before cycle 35. The Cepheid software allowed four optics channels to be monitored in real-time simultaneously. DNA from near-neighbors and no template were used as negative controls. The optimized real-time protocol was evaluated using isolated DNA from 13 *B*. *mallei* isolates, 275 *B*. *pseudomallei* isolates, 11 *B*. *thailandensis* isolates, and 15 genetic near-neighbors (S1 Table).

### Validation of the Multiplex Assay

The identities of the bacterial strains used in this study were verified by both cellular fatty acid (CFA) profiles and previously published molecular assays. CFA analyses were performed using the Sherlock Microbial Identification System (MIDI). CFAs were extracted, methylated, and processed on a 6890N Network GC System (Agilent Technologies), and the data analyzed using Sherlock, version 6.1 software. The GC-FAME profiles were compared to the Rapid Bioterrorism library (RBTR3) and given a match and similarity index.

To further verify the validity of the multiplex assay and ensure the correct identification of *B*. *mallei*, *B*. *pseudomallei*, and *B*. *thailandensis*, real-time PCR assays were used. Adapted versions of the assays developed by U'Ren *et al*.[74] and Thibault *et al*.[25] were used on all samples listed in this study. The primer sequences employed were identical to those reported by U'Ren *et al*. [74], for detection and differentiation of *B*. *mallei* and *B*. *pseudomallei*. U'Ren *et al*'s duplex procedure [74] was used by mixing 1x Hot Start Mix RTG Master Mix (GE Healthcare), 375 nM of each of the *B*. *mallei* and *B*. *pseudomallei* primers and probes, 2 μL of target DNA, and PCR-grade H_2_O to 25 μL. The reactions were then run in the SmartCycler II with the following cycling conditions: 95°C for 150 s followed by 40 cycles of 95°C for 15 s, then 60°C for 45 s. Since a real-time PCR assay is currently unavailable for *B*. *thailandensis*-specific detection, Thibault *et al*'s test [25] was used to detect *B*. *thailandensis* by adding 1x Hot Start Mix RTG Master Mix (GE Healthcare), 500 nM of each *BpSCU2* primer and 200 nm probe, 2 μL of target DNA, and PCR-grade H_2_O to 25 μL. The reactions were then run in the SmartCycler II with the following cycling conditions: 95°C for 150 s followed by 35 cycles of 95°C for 20 s, then 56°C for 60 s. If a sample was negative by U'Ren *et al's* assay [74] and positive by Thibault *et al's* assay [25], it was considered positive for *B*. *thailandensis*.

## Results

### Sensitivity and Specificity Testing

Initial sensitivity and specificity of each primer was evaluated in separate tubes using SYBR Green to detect amplification. All assays except for the *fliC* test yielded threshold amplification in the presence of DNA for their respective *Burkholderia* species. Due to consistent amplification of *B*. *vandii* by the *fliC* primers, a *fliC* probe was designed to exclude *B*. *vandii* (S1 Fig). Having established that the primers were highly specific to their respective DNA targets, SYBR Green was replaced with four specific dual-labeled hydrolysis probes for *B*. *mallei*, *B*. *pseudomallei*, *B*. *thailandensis*, and the common *B*. *pseudomallei*, *B*. *mallei*, and *B*. *thailandensis* group which further increased specificity while maintaining sensitivity to the respective *Burkholderia* species. This also allowed the generation of a multiplexed assay in a single-tube format. All isolates were tested and signal thresholds were exceeded only when DNA for a specific species was present, indicating target sensitivity and specificity.

Of the 314 isolates examined in this study, two purported *B*. *pseudomallei* isolates produced atypical results (S1 Table). These isolates were undetected by the *orf11* and *fliC* assays and both strains tested negative by the previously published assays used in this study [25, 74]. These two isolates were purported to be *B*. *pseudomallei* strains 135 and MSHR 1816. These isolates, received from personal collections of PHE and RDH, were identified as *B*. *pseudomallei* through culture methods. MIDI results from both isolates did not correlate with culture methods, and sequencing data indicated they were not species of the *B*. *pseudomallei* complex. Therefore, against the remaining 312 isolates, all four assays developed in this study were both 100% sensitive and specific for species detection and differentiation.

Since no one physical repository of bacterial DNA samples is complete, we wished to further validate the assays using bioinformatic methods. Specifically, all fully sequenced *B*. *mallei* (14), *B*. *pseudomallei* (52), *B*. *thailandensis* (10), *B*. *oklahomensis* (2), and *B*. *humptydooensis* (2) strains along with single isolates of *B*. *multivorans*, *B*. *cenocepacia*, *B*. *ubonensis*, *B*. *vietnamiensis*, *B*. *dolosa*, and *B*. *cepacia* strains found in NCBI GenBank were compared with our primer/probe sets (S3 Table). Fully sequenced strains from the *Burkholderia* A, B, and C clades were not available. The NCBI Blastn results indicate that the two publicly available strains of *B*. *oklahomensis*, members of the *B*. *pseudomallei* complex, are predicted to be detected by the *fliC* assay. In addition, NCBI BLASTn results indicates the identity values of *B*. *humptydooensis* MSMB 121, another member of the *B*. *pseudomallei* complex, were close to 100% for the *fliC* oligos, but the expected values were sporadic with the forward, reverse, and probe, respectively having values of 19, 22, and 0.067. These values strongly suggest the *fliC* assay would not identify this species. Therefore, this isolate was predicted to be undetected by the *fliC* assay. Our primer/probe sets were found to be 100% specific to the selected NCBI GenBank sequences (S3 Table). Eighteen *B*. *pseudomallei*, three *B*. *thailandensis*, and two *B*. *oklahomensis* isolates have a nucleotide mismatch with oligos used in the *orf11* assay (S3 and S4 Tables) and/or the *fliC* assay (S3 and S5 Tables). Single nucleotide mismatches sometimes allow correct primer/probe binding and sometimes prevent correct primer/probe binding [83–85], therefore these mismatches were further investigated using IDT's DNA Thermodynamic & Hybridization software (S2 and S3 Figs). This software predicts the potential binding success of a mismatch oligo at various temperatures. A fraction of duplex (FoD) value close to 1.0 indicates a higher likelihood of successful binding, and mismatch FoD values generated from the assays ranged from 0.926 to 0.997 (S4 and S5 Tables). It was also shown that using the specific temperature and salt conditions of the singleplex assays, these isolates would very likely still be identified correctly by all four assays (S2 and S3 Figs). In addition, two of the sixteen *B*. *pseudomallei* strains containing mismatches with the *orf11* assay were previously tested by the multiplex assay, MSHR 146 and PHLS 112 (PHE 112), and both isolates tested positive on the *B*. *pseudomallei* specific and *fliC* assays. Therefore, the sensitivity and specificity of all four assays developed in this study appear to be 100% against the 314 strains in our collection and 84 strains with bioinformatic results. However, if in the future it was determined that the present primers/probes do not specifically detect new isolates, then a slight modification including degenerate bases would likely correct this situation, as is commonly performed [86, 87].

### Limit of Detection Testing

**Fig 1:**
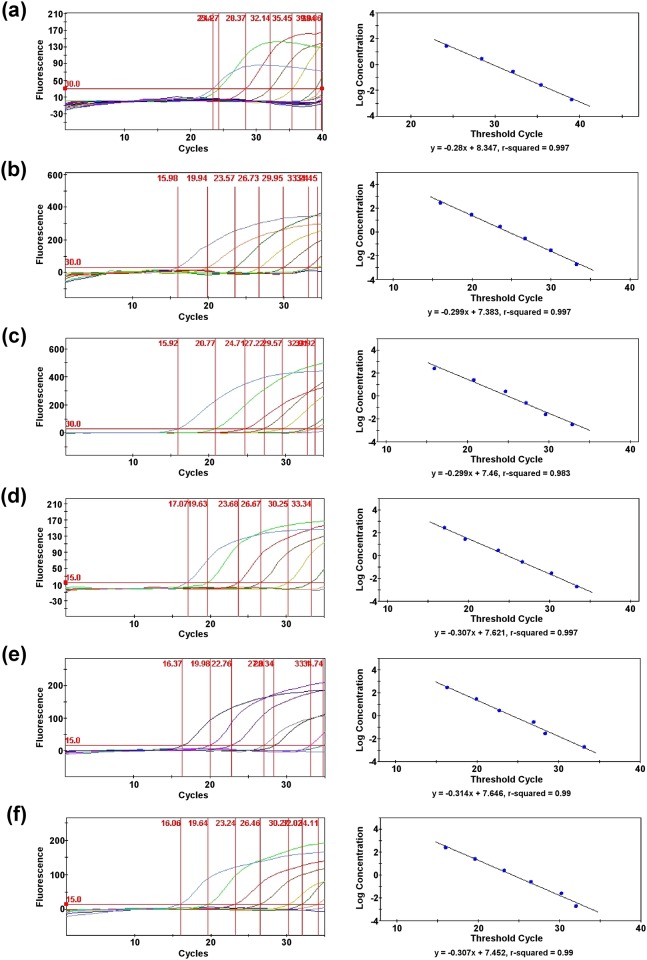
Sensitivity of the singleplex assays. Detection limits of the singleplex assays and standard curves derived from serial dilutions of purified genomic DNAs for some species of the *B*. *pseudomallei* complex. (a) 16.5 kDa assay for *B*. *mallei*, (b) *orf11* assay for *B*. *pseudomallei*, (c) 70 kDa for *B*. *thailandensis*, (d) *fliC* assay for *B*. *mallei*, (e) *fliC* assay for *B*. *pseudomallei*, and (f) *fliC* assay for *B*. *thailandensis*.

**Fig 2:**
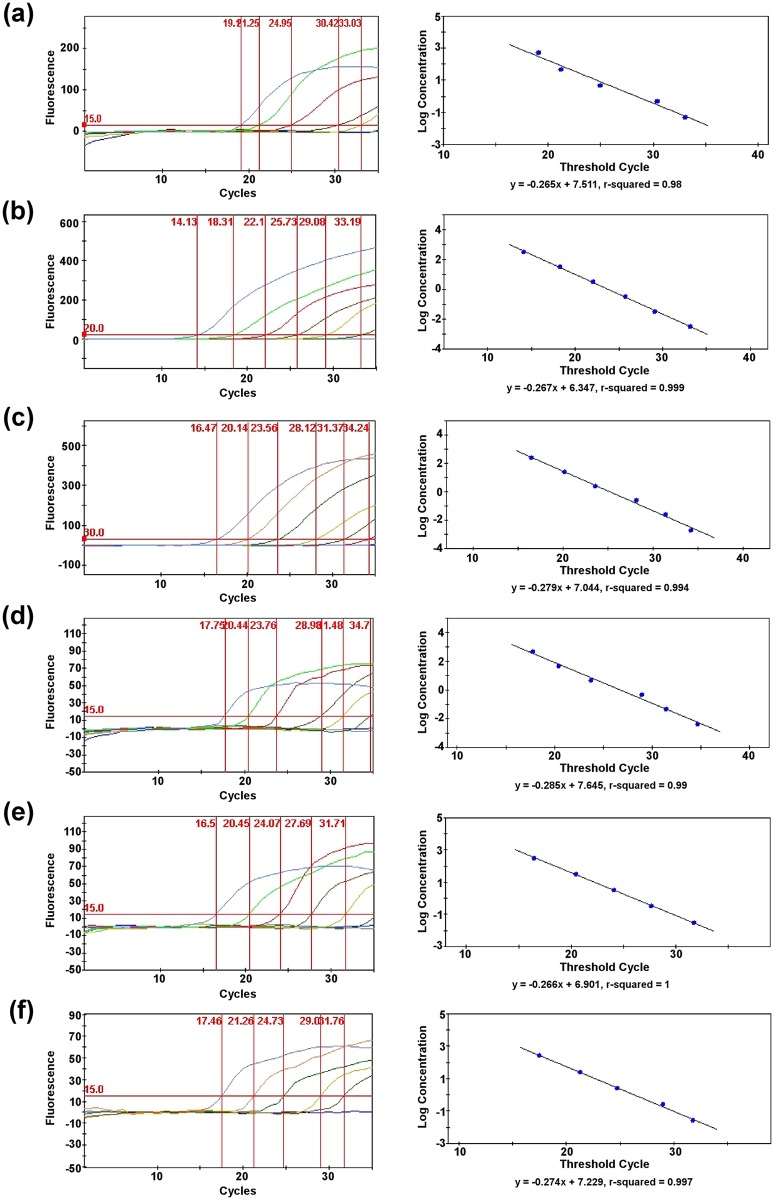
Sensitivity of the multiplex assay. Detection limits of the quadruplex assay and standard curves derived from serial dilutions of purified genomic DNAs for some species of the *B*. *pseudomallei* complex. (a) 16.5 kDa assay for *B*. *mallei*, (b) *orf11* assay for *B*. *pseudomallei*, (c) 70 kDa assay for *B*. *thailandensis*, (d) *fliC* assay for *B*. *mallei*, (e) *fliC* assay for *B*. *pseudomallei*, and (f) *fliC* assay for *B*. *thailandensis*.

For each *Burkholderia* species targeted in the multiplex assay, serial dilutions were made of the purified genomic DNAs, and a genomic equivalent (GE) calculation was determined. For the species-specific singleplex assays, the threshold detection limits were at least 977.2 fg (158 GE) for *B*. *mallei*, 316.16 fg (~40 GE) for *B*. *pseudomallei*, and 520 fg (~72 GE) for *B*. *thailandensis* (Fig 1). For the *fliC* singleplex assay, the threshold sensitivities were at least 4.886 pg (788 GE) for *B*. *mallei*, 316.16 fg (~40 GE) for *B*. *pseudomallei*, and 520 fg (~72 GE) for *B*. *thailandensis*. For the quadruplex assay, the detection limits of the species-specific targets were at least 48.86 pg (7,880 GE) for *B*. *mallei*, 3.1616 pg (404 GE) for *B*. *pseudomallei*, and 2.6 pg (358 GE) for *B*. *thailandensis* (Fig 2). For the *fliC* component of the multiplex assay, the threshold detection limits were at least 4.886 pg (788 GE) for *B*. *mallei*, 31.616 pg (4,040 GE) for *B*. *pseudomallei*, and 26 pg (3,580 GE) for *B*. *thailandensis*.

### Comparing the Multiplex Assay

The GC-FAME results showed identification discrepancies for some isolates used in this study. The GC-FAME RBTR3 library was unable to distinguish the fatty acid profiles of some *B*. *pseudomallei* and *B*. *thailandensis* isolates. In addition, four *B*. *pseudomallei* MSHR isolates were identified as *B*. *mallei* or *B*. *cenocepacia/B*. *pyrrocinia/B*. *ambifaria* (S1 Table). In short, the GC-FAME sensitivity and specificity for *B*. *mallei* was respectively 100% (13/13) and 98.6% (282/286) while the sensitivity and specificity for *B*. *pseudomallei* was respectively 98.5% (269/273) and 57.7% (15/26). All of the *B*. *thailandensis* isolates used in this study were identified as *B*. *pseudomallei*. Two previously published real-time PCR tests described by U'Ren *et al*. [74] and Thibault *et al*. [25] were performed to compare with the results obtained by our real-time PCR assay. The results of these previously published tests correlated exactly with the multiplex assay. U'Ren *et al's* duplex assay [74] correctly identified the *B*. *mallei* and *B*. *pseudomallei* isolates used in this study. The two presumed *B*. *pseudomallei* strains were also negative by U'Ren *et al's* assay [74]. In addition, Thibault *et al's* assay [25] was positive for the *B*. *thailandensis* strains used in this study.

## Discussion

Glanders and melioidosis are significant diseases with high mortality rates, if left untreated. The difficulty in identifying the species of the *B*. *pseudomallei* complex and the bioweapon potential of some of these species necessitated the development of a more rapid and accurate detection method. Having access to accurate PCR assays with minimal diagnostic times could decrease mortality rates. Although at present there is limited access to PCR in some endemic areas, access is likely to continue increasing in the future.

Although PCR-based procedures have revolutionized microbial detection, they do have limitations. Both false positives and false negatives can occur [88, 89]. Some of this can be minimized with good technique, and some can be minimized with good assay design. It is possible, especially in newly emerging pathogens, for mutations to occur in the gene of interest, which can then compromise the test [90]. This issue can be overcome by developing multiplex assays.

Some studies showing false positive results have reported sensitivities beyond culture for clinical and environmental samples, that correlate with serological, radiological, and/or additional PCR-based assays [59]. Detection of non-viable *B*. *pseudomallei* in clinical samples from confirmed melioidosis patients has also been observed [91]. Although PCR false positives are considered inaccurate, they may represent the presence of unculturable *Burkholderia* species in clinical and environmental samples, and therefore may be more accurate than culture due to the inability of some bacteria to grow via existing culture methods. One study observed that 45% of septicemic melioidosis patients exhibited less than 1 CFU/mL of *B*. *pseudomallei* in their blood samples [92], which reinforces the need for sensitive testing methods such as PCR-based assays. Another possibility for some false positive results in unknown samples may be due to near-neighbors with similar DNA sequence to the targets, thus highlighting the need for multiple targets in a molecular assay.

In a recent review of all real-time PCR assays developed for detection and differentiation of *B*. *pseudomallei*, *B*. *mallei*, and *B*. *thailandensis*, it was observed that mutations in the form of gene deletions have affected assay sensitivity in only two studies [59]. Three strains from these studies lacked some virulence loci, indicating possible avirulent variants of *B*. *mallei* and *B*. *pseudomallei*. From these limited examples, it appears mutations within PCR-targeted genes have not significantly compromised the reliability of PCR-based tests for the detection of *B*. *pseudomallei* complex organisms. It is widely accepted that *B*. *mallei* evolved as a strict pathogen from *B*. *pseudomallei* [31, 32]. Despite the major evolutionary changes between these two species, they continue to be identified as a complex in several PCR-based methods.

Real-time PCR is able to resolve some of the limitations previously described, because of the versatility and additional specificity of the internal probe. The probe technology allows for simultaneous detection of multiple targets, which can overcome the problem of potential gene mutations at a single locus. Overall, real-time PCR assays are generally considered to have a large dynamic range, low inter-assay variation, and high reliability [93].

GC-FAME analysis identified *B*. *pseudomallei* MSHR 2394 as *B*. *cenocepacia*/*B*. *pyrrocinia*/*B*. *ambifaria*, and *B*. *pseudomallei* MSHR strains 1912, 1954, and 1986 as *B*. *mallei*. GC-FAME analysis also identified the purported *B*. *pseudomallei* 135 as *B*. *mallei* while the purported *B*. *pseudomallei* MSHR 1816 produced no match. A 98% sensitivity and an 83.3% specificity for *B*. *pseudomallei* strains was reported by GLC-FAME analysis [48]. Our results confirmed Inglis *et al*'s conclusion [48] that a PCR-based method was more accurate and precise than GC-FAME methods.

The multiplex assay described here is the first of its kind that can detect and differentiate between *B*. *mallei*, *B*. *pseudomallei*, and *B*. *thailandensis* in a single-tube format. This test has overcome several PCR-based drawbacks related to sensitivity and specificity. The negative results produced by the two purported *B*. *pseudomallei* isolates increases the reliability of the multiplex assay, in that it displayed a higher sensitivity than culture. The multiplex assay may help future research determine if PCR-based assays could replace culture methods, since the *fliC* target functions as a second confirmation within the assay. If a species-specific target in the multiplex assay fails, the *fliC* target detects the entire *B*. *pseudomallei*, *B*. *mallei*, *B*. *thailandensis*, and possibly *B*. *oklahomensis* group and can still detect and differentiate by creating a unique amplification profile.

It was reported that *B*. *sordidicola* CCUG 49583 and two *B*. *thailandensis* strains were detected by Thibault *et al*'s *B*. *pseudomallei* specific *orf11* assay [65]. The *orf11* target used in the multiplex assay was negative for the same *B*. *sordidicola* strain, and was also negative for the 11 *B*. *thailandensis* strains evaluated in this study. The high sensitivity of real-time PCR also proved to be beneficial during the development of the *fliC* probe. Although each *fliC* primer differed from the *B*. *vandii* sequence by one nucleotide, amplification was observed in this species. The added sensitivity of the internal probe made it possible to exclude *B*. *vandii* while maintaining positive detection of the isolates in the *B*. *pseudomallei*, *B*. *mallei*, *B*. *thailandensis*, and possibly *B*. *oklahomensis* group. The multiplex assay results agreed with those of previously published tests, and also produced faster results than current multiplex PCR methods because of its single-tube format design for detecting and differentiating several members of the *B*. *pseudomallei* complex.

The multiplex assay is both 100% sensitive and specific using purified DNA from the isolates examined in this study; although it is always possible that other isolates exist, or may emerge, with differing sequences that would not be detected. The multiplex assay is rapid, demonstrates sensitivity and specificity beyond culture and GC-FAME methods, and is robust due to its ability to still detect and differentiate, by creating a unique amplification profile, if a species-specific component of the multiplex assay fails. This test is also the only PCR assay currently available that is capable of specifically detecting and differentiating *B*. *pseudomallei*, *B*. *mallei*, and *B*. *thailandensis* in a single-tube format. For these reasons, this assay could prove useful as a rapid, sensitive, and economical tool in the detection and or differentiation for several species within the *B*. *pseudomallei* complex. If the newly discovered *B*. *oklahomensis*, *B*. *humptydooensis*, and *Burkholderia* clades A, B, and C prove to be clinically important, the multiplex assay described here could likely be adapted to include additional targets for these new species of the *B*. *pseudomallei* complex.

## Supporting Information

S1 Fig Development of the *fliC* probe. The *fliC* probe was developed based on sequence information of the *B*. *pseudomallei* complex and *B*. *vandii* with their corresponding accession numbers.
(PDF)

S2 Fig Properties of *B*. *pseudomallei orf11* assay mismatches. NCBI BLASTn results for some *B*. *pseudomallei* strains indicate the *orf11* assay contained four different mismatches that were found in the forward primer (A-B), reverse primer (C) and/or the probe (D). Integrated DNA Technologies' DNA Thermodynamics & Hybridization software was used to analyze the oligo sequence with the corresponding sequence shown with the mismatch being indicated by letters in red. T_m_, Gibbs Energy, Enthalpy, and Entropy for each match or mismatch is shown with the salt concentrations used in the *orf11* assay as indicated. The hybridization profiles are also shown for the exact match (blue) and mismatch (red). Refer to S4 Table for a summary of the mismatch profiles of the *orf11* assay against *B*. *pseudomallei* isolates from NCBI GenBank.
(PDF)

S3 Fig Properties of the *fliC* assay mismatches. NCBI BLASTn results indicate the *fliC* assay contained three different mismatches that were found in the probe for some *B*. *pseudomallei* (A-B) and for some *B*. *thailandensis* and *B*. *oklahomensis* strains (C-D). The third mismatch was also found in the forward primer for *B*. *oklahomensis* strains (E-F). Integrated DNA Technologies' DNA Thermodynamics & Hybridization software was used to analyze the probe or primer sequence with the corresponding sequence shown with the mismatch being indicated by letters in red. T_m_, Gibbs Energy, Enthalpy, and Entropy for each match or mismatch is shown with the salt concentrations used in the *fliC* assay as indicated. The hybridization profiles are also shown for the exact match (blue) and mismatch (red). The software is unable to calculate degenerate bases. Therefore, both bases were calculated to ensure accuracy of a degenerate base with (A and C) containing the guanine base at position 21 of the probe, (B and D) adenine base at position 21 of the probe, (E) cytosine at position 12 of the forward primer, and (F) thymine at position 12 of the forward primer. Refer to S5 Table for a summary of the mismatch profiles of the *fliC* assay against *B*. *pseudomallei* complex isolates from NCBI GenBank.
(PDF)

S1 Table Results of the quadruplex assay for individual bacterial isolates. (PDF)

S2 Table Number and origin of *B*. *pseudomallei* complex strains evaluated. (PDF)

S3 Table Suspected multiplex assay results based on NCBI BLASTn and DNA Thermodynamics & Hybridization software. The multiplex assay was evaluated against all completed sequences of *B*. *mallei*, *B*. *pseudomallei*, *B*. *thailandensis*, *B*. *oklahomensis*, *B*. *humptydooensis* strains along with a few other *Burkholderia* near-neighbors from the NCBI GenBank database.
(PDF)

S4 Table Summary of the mismatch profiles of the *orf11* assay against *B*. *pseudomallei* isolates from NCBI GenBank. Fraction of duplex values were calculated using DNA Thermodynamic & Hybridization software from Integrated DNA Technologies to determine the binding efficiency of an oligo when single nucleotide polymorphisms are present. A FoD value closer to 1 indicates a higher likelihood of proper binding.
(PDF)

S5 Table Summary of the mismatch profiles of the *fliC* assay against *B*. *pseudomallei* complex isolates from NCBI GenBank. Fraction of duplex values were calculated using DNA Thermodynamics & Hybridization software from Integrated DNA Technologies to determine the binding efficiency of an oligo when single nucleotide polymorphisms are present. A FoD value closer to 1 indicates a higher likelihood of proper binding. The software is unable to calculate degenerate bases; therefore, both bases were calculated and the average FoD is presented in this table.
(PDF)
