# Supplementary material for: A Quadruplex Real-Time PCR Assay for the Rapid Detection and Differentiation of the Most Relevant Members of the B. pseudomallei Complex: B. mallei, B. pseudomallei, and B. thailandensis
Source: PLoS One. 2016 Oct 13;11(10):e0164006. doi: 10.1371/journal.pone.0164006 (PMC5063335; doi:10.1371/journal.pone.0164006)
Supplement: S1 Fig — The fliC probe was developed based on sequence information of the B. pseudomallei complex and B. vandii with their corresponding accession numbers. (PDF) [file pone.0164006.s001.pdf]

```
                                                    20                                      40
B. mallei (AF098793.1)           ACGGTCAACA ATCTGCAGGC AACGCTCGGC GCGGCGCAAA ACCGCTTCAC 50
B.pseudomallei (BX571965.1)      ACGGTCAACA ATCTGCAGGC AACGCTCGGC GCGGCGCAAA ACCGCTTCAC 50
B.thailandensis (AF081500.1)     ACGGTCAACA ACCTGCAGGC AACGCTCGGC GCGGCGCAGA ACCGCTTCAC 50
B.vandii ATCC 51545              ACGATCAACA ACCTGCAGGC AACGCTCGGT GCAACCCAGA ACCGACTGCA 50
Forward Primer                   ACGGTCAACA ATCTGCAGGC AA-------- ---------- ---------- 22
Probe                            ---------- ---------- ---------- ---------- ---------- -
Reverse Primer                   ---------- ---------- ---------- ---------- ---------- -

                                          60                    80                    100
B. mallei (AF098793.1)           CGCGATCGCG ACGACGCAGC AAGCCGGCTC GAACAACCTC GCGCAGGCGC 100
B.pseudomallei (BX571965.1)      CGCGATCGCG ACGACGCAGC AAGCCGGCTC GAACAACCTC GCGCAGGCGC 100
B.thailandensis (AF081500.1)     CGCGATCGCG ACCACGCAGC AAGCCGGCTC GAACAACCTC GCGCAAGCGC 100
B.vandii ATCC 51545              GGCCATCGCG CAAACGCAGC AAGCCAACGC GACGAACCTG TCGTCGGCTC 100
Forward Primer                   ---------- ---------- ---------- ---------- ---------- 22
Probe                            ---------- ---------- -----GGCTC GAACAACCTC GCGCARG--- 22
Reverse Primer                   ---------- ---------- ---------- ---------- ---------- -

                                                    120                   140
B. mallei (AF098793.1)           AATCGCAAAT CCAGAGCGCG GACTTTGCTC AGGAAACCGC GAAC 144
B.pseudomallei (BX571965.1)      AATCGCAAAT CCAGAGCGCG GACTTTGCTC AGGAAACCGC GAAC 144
B.thailandensis (AF081500.1)     AATCGCAAAT CCAGAGCGCG GACTTCGCTC AGGAAACCGC GAAC 144
B.vandii ATCC 51545              AATCGCAAAT CCAGAGCGCC GACTTCGCGC AGGAAACCGC GAAC 144
Forward Primer                   ---------- ---------- ---------- ---------- ---- 22
Probe                            ---------- ---------- ---------- ---------- ---- 22
Reverse Primer                   ---------- ---------- GACTTTGCTC AGGAAACCGC GAAC 24
```
