# Supplementary material for: A Quadruplex Real-Time PCR Assay for the Rapid Detection and Differentiation of the Most Relevant Members of the B. pseudomallei Complex: B. mallei, B. pseudomallei, and B. thailandensis
Source: PLoS One. 2016 Oct 13;11(10):e0164006. doi: 10.1371/journal.pone.0164006 (PMC5063335; doi:10.1371/journal.pone.0164006)

A

Mismatch sequence:

5'- A A C A C T G A C A A G T G G C C C T A T G G A -3'
3'- T T G T G A C T G T T C G C C G G G A T A C C T -5'

Exact match sequence:

5'- A A C A C T G A C A A G T G G C C C T A T G G A -3'
3'- T T G T G A C T G T T C A C C G G G A T A C C T -5'

	T_m [°C]	Gibbs Energy (ΔG_{37}) [kcal/mol]	Enthalpy (ΔH) [kcal/mol]	Entropy (ΔS) [cal/(K·mol)]
**Mismatch**	67.10	-24.48	-174.90	-485.00
**Exact match**	68.84	-26.07	-183.30	-506.95
**Difference**	1.73	-1.59	-8.40	-21.96

Duplex Length: 24

Oligo Conc: 0.45 μM
Target Conc: 0.00 μM
Na^+, K^+ Conc: 50.00 mM
Mg^{2+} Conc: 1.50 mM
dNTPs Conc: 0.20 mM

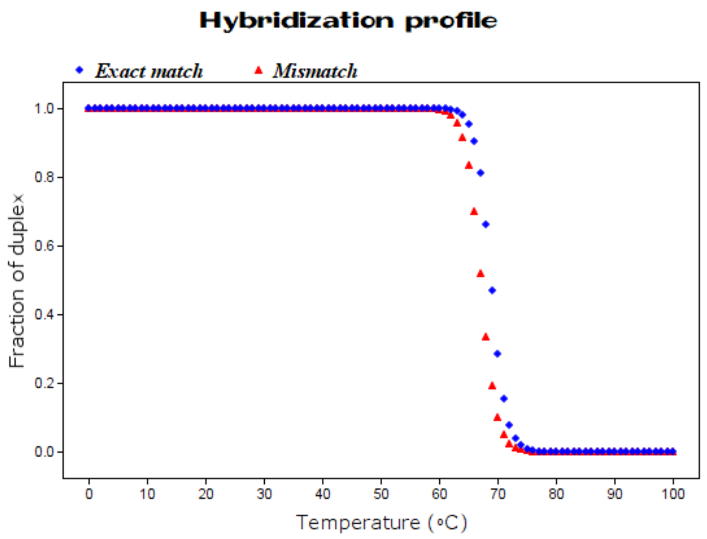

B

Mismatch sequence:

5'- A A C A C T G A C A A G T G G C C C T A T G G A -3'
3'- T T G T G A C T G T T C A C C G G G A T A G C T -5'

Exact match sequence:

5'- A A C A C T G A C A A G T G G C C C T A T G G A -3'
3'- T T G T G A C T G T T C A C C G G G A T A C C T -5'

	T_m [°C]	Gibbs Energy (ΔG_{37}) [kcal/mol]	Enthalpy (ΔH) [kcal/mol]	Entropy (ΔS) [cal/(K·mol)]
**Mismatch**	64.33	-22.78	-170.10	-475.01
**Exact match**	68.84	-26.07	-183.30	-506.95
**Difference**	4.51	-3.29	-13.20	-31.95

Duplex Length: 24

Oligo Conc: 0.45 μM
Target Conc: 0.00 μM
Na^+, K^+ Conc: 50.00 mM
Mg^{2+} Conc: 1.50 mM
dNTPs Conc: 0.20 mM

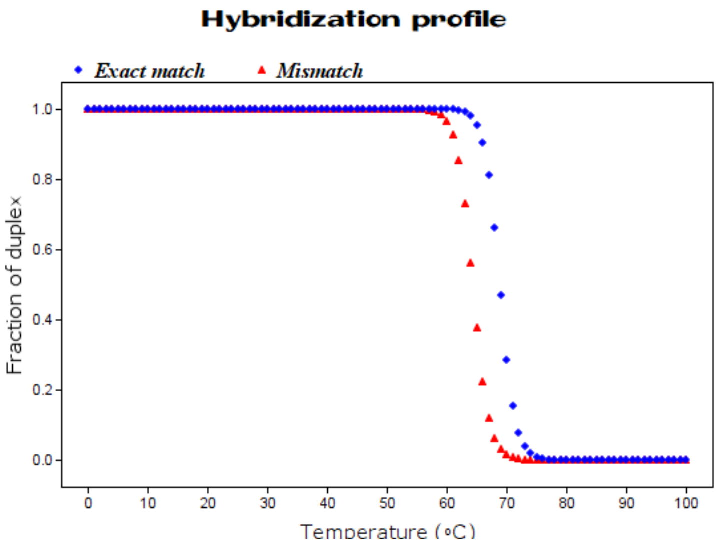

C

Mismatch sequence:

5'- T C C G A T C G G T T T C G A A T A A C G G G T -3'
3'- A G G C C A G C C A A A G C T T A T T G C C C A -5'

Exact match sequence:

5'- T C C G A T C G G T T T C G A A T A A C G G G T -3'
3'- A G G C T A G C C A A A G C T T A T T G C C C A -5'

	T_m [°C]	Gibbs Energy (ΔG_{37}) [kcal/mol]	Enthalpy (ΔH) [kcal/mol]	Entropy (ΔS) [cal/(K·mol)]
**Mismatch**	67.26	-23.52	-163.30	-450.69
**Exact match**	68.77	-26.58	-189.20	-524.32
**Difference**	1.51	-3.06	-25.90	-73.63

Duplex Length: 24

Oligo Conc: 0.45 µM Na^+, K^+ Conc: 50.00 mM   dNTPs Conc: 0.20 mM
Target Conc: 0.00 µM Mg^{2+} Conc: 1.50 mM

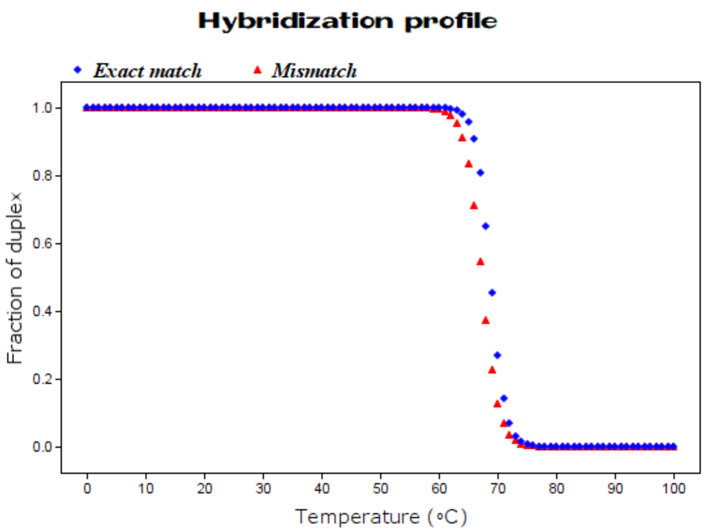

D

Mismatch sequence:

5'- A C G A T C T C C G A G A A C G C A C T G A A C A -3'
3'- T G T T A G A G G C T C T T G C G T G A C T T G T -5'

Exact match sequence:

5'- A C G A T C T C C G A G A A C G C A C T G A A C A -3'
3'- T G C T A G A G G C T C T T G C G T G A C T T G T -5'

	T_m [°C]	Gibbs Energy (ΔG_{37}) [kcal/mol]	Enthalpy (ΔH) [kcal/mol]	Entropy (ΔS) [cal/(K·mol)]
**Mismatch**	66.79	-25.40	-186.30	-518.77
**Exact match**	70.40	-28.49	-199.70	-552.02
**Difference**	3.61	-3.09	-13.40	-33.25

Duplex Length: 25

Oligo Conc: 0.40 µM Na^+, K^+ Conc: 50.00 mM   dNTPs Conc: 0.20 mM
Target Conc: 0.00 µM Mg^{2+} Conc: 1.50 mM

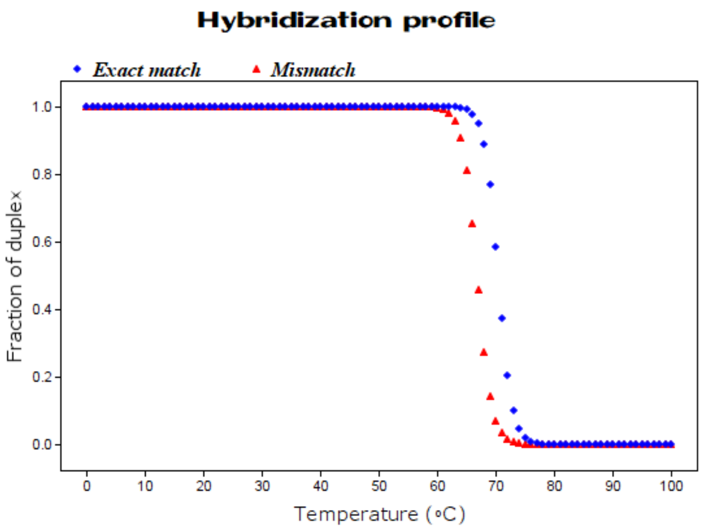

Supplement: S2 Fig — NCBI BLASTn results for some B. pseudomallei strains indicate the orf11 assay contained four different mismatches that were found in the forward primer (A-B), reverse primer (C) and/or the probe (D). Integrated DNA Technologies' DNA Thermodynamics & Hybridization software was used to analyze the oligo sequence with the corresponding sequence shown with the mismatch being indicated by letters in red. Tm, Gibbs Energy, Enthalpy, and Entropy for each match or mismatch is shown with the salt concentrations used in the orf11 assay as indicated. The hybridization profiles are also shown for the exact match (blue) and mismatch (red). Refer to S4 Table for a summary of the mismatch profiles of the orf11 assay against B. pseudomallei isolates from NCBI GenBank. (PDF) [file pone.0164006.s002.pdf]
