# Supplementary material for: A Quadruplex Real-Time PCR Assay for the Rapid Detection and Differentiation of the Most Relevant Members of the B. pseudomallei Complex: B. mallei, B. pseudomallei, and B. thailandensis
Source: PLoS One. 2016 Oct 13;11(10):e0164006. doi: 10.1371/journal.pone.0164006 (PMC5063335; doi:10.1371/journal.pone.0164006)

A

Mismatch sequence:

5'- G G C T C G A A C A A C C T C G C G C A G G -3'
3'- C C G A G T T T G T T G G A G C G C G T C C -5'

Exact match sequence:

5'- G G C T C G A A C A A C C T C G C G C A G G -3'
3'- C C G A G C T T G T T G G A G C G C G T C C -5'

	T_m [°C]	Gibbs Energy (ΔG_{37}) [kcal/mol]	Enthalpy (ΔH) [kcal/mol]	Entropy (ΔS) [cal/(K·mol)]
**Mismatch**	69.89	-25.30	-169.20	-463.97
**Exact match**	73.75	-28.42	-182.60	-497.11
**Difference**	3.86	-3.12	-13.40	-33.14

Duplex Length: 22

Oligo Conc: 0.40 μM | Na^+, K^+ Conc: 50.00 mM | dNTPs Conc: 0.20 mM
Target Conc: 0.00 μM | Mg^{2+} Conc: 1.50 mM

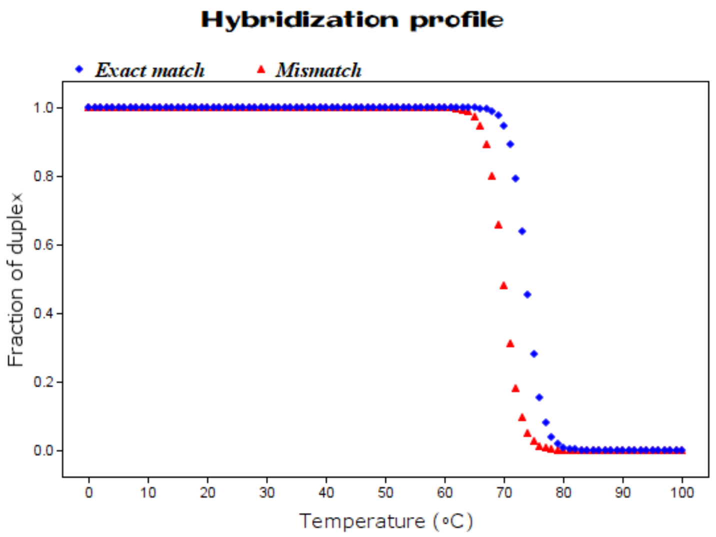

B

Mismatch sequence:

5'- G G C T C G A A C A A C C T C G C G C A A G -3'
3'- C C G A G T T T G T T G G A G C G C G T T C -5'

Exact match sequence:

5'- G G C T C G A A C A A C C T C G C G C A A G -3'
3'- C C G A G C T T G T T G G A G C G C G T T C -5'

	T_m [°C]	Gibbs Energy (ΔG_{37}) [kcal/mol]	Enthalpy (ΔH) [kcal/mol]	Entropy (ΔS) [cal/(K·mol)]
**Mismatch**	67.83	-24.36	-169.10	-466.67
**Exact match**	71.77	-27.48	-182.50	-499.84
**Difference**	3.95	-3.11	-13.40	-33.17

Duplex Length: 22

Oligo Conc: 0.40 μM | Na^+, K^+ Conc: 50.00 mM | dNTPs Conc: 0.20 mM
Target Conc: 0.00 μM | Mg^{2+} Conc: 1.50 mM

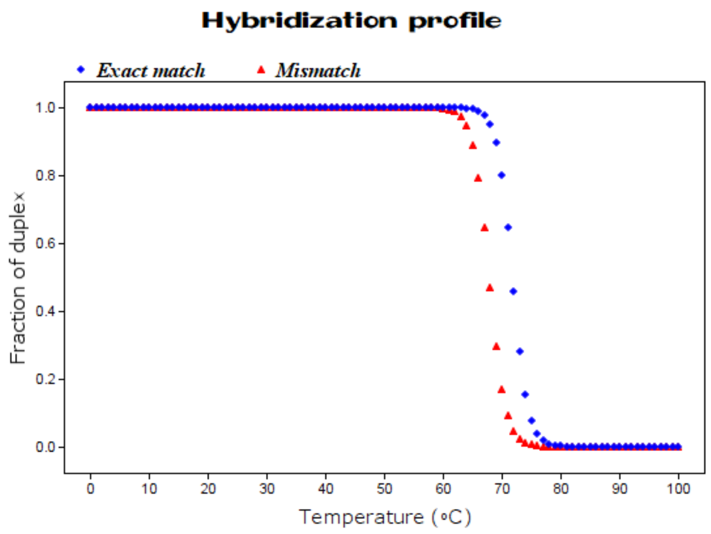

C

Mismatch sequence:

5'- G G C T C G A A C A A C C T C G C G C A G G -3'
3'- C C G A G C T T G T T G G A C C G C G T C C -5'

Exact match sequence:

5'- G G C T C G A A C A A C C T C G C G C A G G -3'
3'- C C G A G C T T G T T G G A G C G C G T C C -5'

	T_m [°C]	Gibbs Energy (ΔG_{37}) [kcal/mol]	Enthalpy (ΔH) [kcal/mol]	Entropy (ΔS) [cal/(K·mol)]
**Mismatch**	68.38	-23.71	-159.20	-436.87
**Exact match**	73.75	-28.42	-182.60	-497.11
**Difference**	5.37	-4.72	-23.40	-60.24

Duplex Length: 22

Oligo Conc: 0.40 µM Na^+, K^+ Conc: 50.00 mM   dNTPs Conc: 0.20 mM
Target Conc: 0.00 µM Mg^{2+} Conc: 1.50 mM

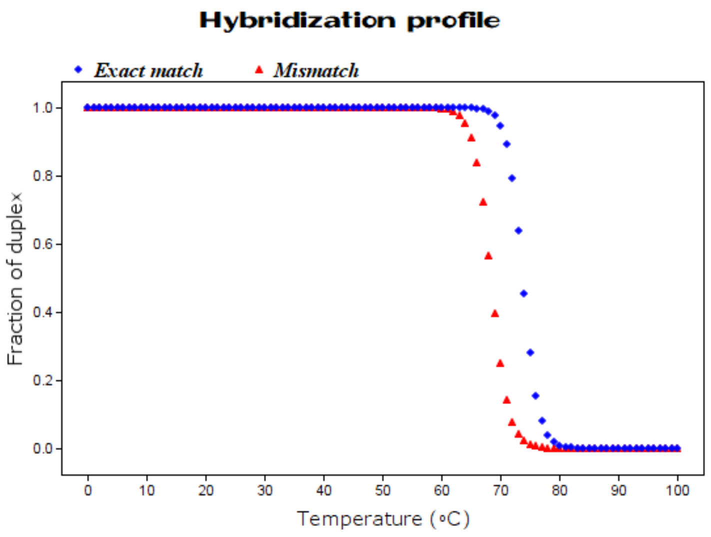

D

Mismatch sequence:

5'- G G C T C G A A C A A C C T C G C G C A A G -3'
3'- C C G A G C T T G T T G G A C C G C G T T C -5'

Exact match sequence:

5'- G G C T C G A A C A A C C T C G C G C A A G -3'
3'- C C G A G C T T G T T G G A G C G C G T T C -5'

	T_m [°C]	Gibbs Energy (ΔG_{37}) [kcal/mol]	Enthalpy (ΔH) [kcal/mol]	Entropy (ΔS) [cal/(K·mol)]
**Mismatch**	66.22	-22.78	-159.10	-439.54
**Exact match**	71.77	-27.48	-182.50	-499.84
**Difference**	5.55	-4.70	-23.40	-60.30

Duplex Length: 22

Oligo Conc: 0.40 µM Na^+, K^+ Conc: 50.00 mM   dNTPs Conc: 0.20 mM
Target Conc: 0.00 µM Mg^{2+} Conc: 1.50 mM

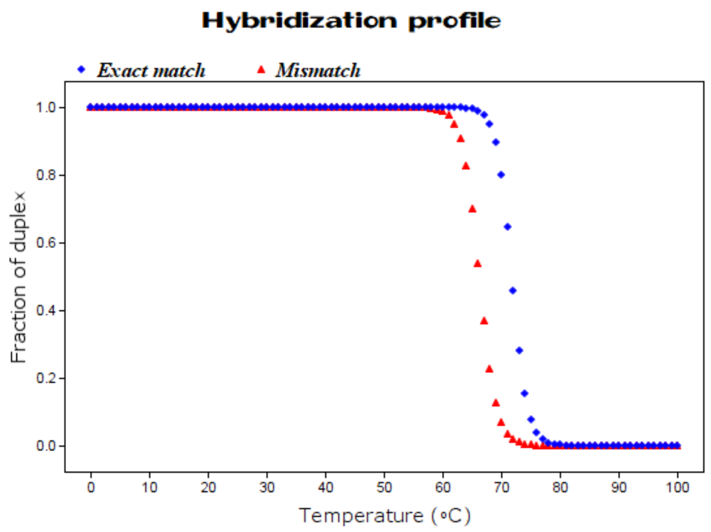

E

Mismatch sequence:

5'- A C G G T C A A C A A C C T G C A G G C A A -3'
3'- T G C C A C T T G T T G G A C G T C C G T T -5'

Exact match sequence:

5'- A C G G T C A A C A A C C T G C A G G C A A -3'
3'- T G C C A G T T G T T G G A C G T C C G T T -5'

	T_m [°C]	Gibbs Energy (ΔG_{37}) [kcal/mol]	Enthalpy (ΔH) [kcal/mol]	Entropy (ΔS) [cal/(K·mol)]
**Mismatch**	67.09	-21.84	-144.30	-394.85
**Exact match**	70.47	-25.95	-173.20	-474.79
**Difference**	3.38	-4.11	-28.90	-79.93

Duplex Length: 22

Oligo Conc: 0.40 µM
Target Conc: 0.00 µM
Na^+, K^+ Conc: 50.00 mM
Mg^{2+} Conc: 1.50 mM
dNTPs Conc: 0.20 mM

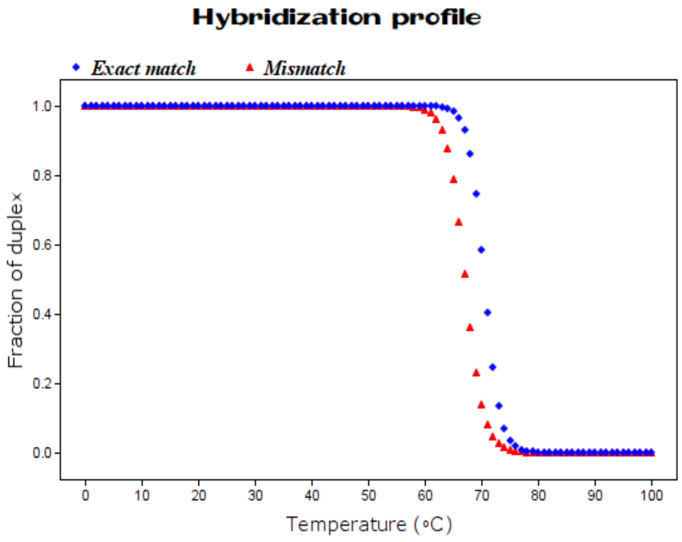

F

Mismatch sequence:

5'- A C G G T C A A C A A T C T G C A G G C A A -3'
3'- T G C C A C T T G T T A G A C G T C C G T T -5'

Exact match sequence:

5'- A C G G T C A A C A A T C T G C A G G C A A -3'
3'- T G C C A G T T G T T A G A C G T C C G T T -5'

	T_m [°C]	Gibbs Energy (ΔG_{37}) [kcal/mol]	Enthalpy (ΔH) [kcal/mol]	Entropy (ΔS) [cal/(K·mol)]
**Mismatch**	64.28	-20.66	-143.30	-395.42
**Exact match**	68.05	-24.75	-172.20	-475.42
**Difference**	3.77	-4.09	-28.90	-80.00

Duplex Length: 22

Oligo Conc: 0.40 µM
Target Conc: 0.00 µM
Na^+, K^+ Conc: 50.00 mM
Mg^{2+} Conc: 1.50 mM
dNTPs Conc: 0.20 mM

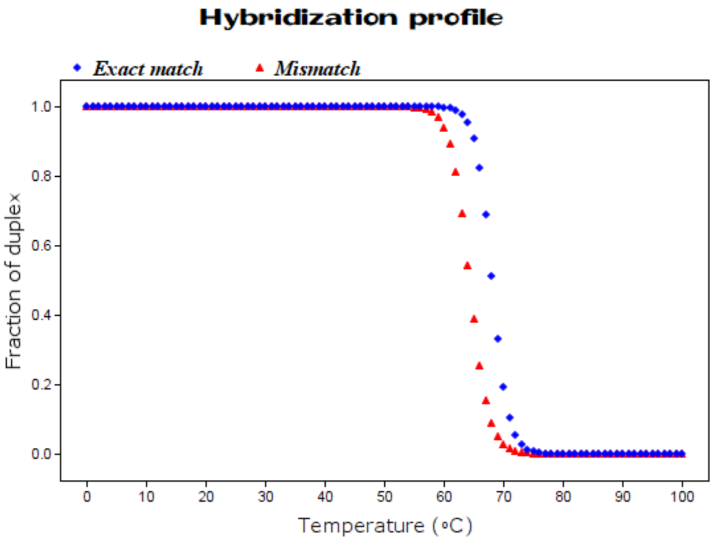

Supplement: S3 Fig — NCBI BLASTn results indicate the fliC assay contained three different mismatches that were found in the probe for some B. pseudomallei (A-B) and for some B. thailandensis and B. oklahomensis strains (C-D). The third mismatch was also found in the forward primer for B. oklahomensis strains (E-F). Integrated DNA Technologies' DNA Thermodynamics & Hybridization software was used to analyze the probe or primer sequence with the corresponding sequence shown with the mismatch being indicated by letters in red. Tm, Gibbs Energy, Enthalpy, and Entropy for each match or mismatch is shown with the salt concentrations used in the fliC assay as indicated. The hybridization profiles are also shown for the exact match (blue) and mismatch (red). The software is unable to calculate degenerate bases. Therefore, both bases were calculated to ensure accuracy of a degenerate base with (A and C) containing the guanine base at position 21 of the probe, (B and D) adenine base at position 21 of the probe, (E) cytosine at position 12 of the forward primer, and (F) thymine at position 12 of the forward primer. Refer to S5 Table for a summary of the mismatch profiles of the fliC assay against B. pseudomallei complex isolates from NCBI GenBank. (PDF) [file pone.0164006.s003.pdf]
