# Supplementary material for: A Quadruplex Real-Time PCR Assay for the Rapid Detection and Differentiation of the Most Relevant Members of the B. pseudomallei Complex: B. mallei, B. pseudomallei, and B. thailandensis
Source: PLoS One. 2016 Oct 13;11(10):e0164006. doi: 10.1371/journal.pone.0164006 (PMC5063335; doi:10.1371/journal.pone.0164006)
Supplement: S1 Table — (PDF) [file pone.0164006.s004.pdf]

Species	Isolate	Source	GC-FAME (RBTR3)	U'Ren *et al.*, (2005) qPCR	Thibault *et al.*, (2004) qPCR	16.5 kDa (Bm)	*Orf11* (Bp)	70 kDa (Bt)	*fliC* (Bp Complex)
B. mallei	Turkey #1	CDC	Bm	Bm	NT	+	-	-	+
B. mallei	85-503	CDC	Bm	Bm	NT	+	-	-	+
B. mallei	86-567-2	CDC	Bm	Bm	NT	+	-	-	+
B. mallei	120	NCTC	Bm	Bm	NT	+	-	-	+
B. mallei	3708	NCTC	Bm	Bm	NT	+	-	-	+
B. mallei	3709	NCTC	Bm	Bm	NT	+	-	-	+
B. mallei	10229	NCTC	Bm	Bm	NT	+	-	-	+
B. mallei	10247	NCTC	Bm	Bm	NT	+	-	-	+
B. mallei	10248	NCTC	Bm	Bm	NT	+	-	-	+
B. mallei	10260	NCTC	Bm	Bm	NT	+	-	-	+
B. mallei	10399	ATCC	Bm	Bm	NT	+	-	-	+
B. mallei	15310	ATCC	Bm	Bm	NT	+	-	-	+
B. mallei	23344	ATCC	Bm	Bm	NT	+	-	-	+
B. thailandensis	E27	UC	Bp	-	+	-	-	+	+
B. thailandensis	E96	UC	Bp	-	+	-	-	+	+
B. thailandensis	E125	UC	Bp	-	+	-	-	+	+
B. thailandensis	E135	UC	Bp	-	+	-	-	+	+
B. thailandensis	E254	NCTC	Bp	-	+	-	-	+	+
B. thailandensis	E255	UC	Bp	-	+	-	-	+	+
B. thailandensis	E263	UC	Bp	-	+	-	-	+	+
B. thailandensis	E264	UC	Bp	-	+	-	-	+	+
B. thailandensis	E275	UC	Bp	-	+	-	-	+	+
B. thailandensis	E286	UC	Bp	-	+	-	-	+	+
B. thailandensis	82172	PHE	Bp	-	+	-	-	+	+
B. pseudomallei	8	PHE	Bp	Bp	NT	-	+	-	+
B. pseudomallei	9	PHE	Bp	Bp	NT	-	+	-	+

**Species**	**Isolate**	**Source**	**GC-FAME (RBTR3)**	**U'Ren *et al.*, (2005) qPCR**	**Thibault *et al.*, (2004) qPCR**	**16.5 kDa (Bm)**	***Orf11* (Bp)**	**70 kDa (Bt)**	***fliC* (Bp Complex)**
B. pseudomallei	14	PHE	Bp	Bp	NT	-	+	-	+
B. pseudomallei	16	PHE	Bp	Bp	NT	-	+	-	+
B. pseudomallei	17	PHE	Bp	Bp	NT	-	+	-	+
B. pseudomallei	18	PHE	Bp	Bp	NT	-	+	-	+
B. pseudomallei	19	PHE	Bp	Bp	NT	-	+	-	+
B. pseudomallei	20	PHE	Bp	Bp	NT	-	+	-	+
B. pseudomallei	24	PHE	Bp	Bp	NT	-	+	-	+
B. pseudomallei	25	PHE	Bp	Bp	NT	-	+	-	+
B. pseudomallei	31	PHE	Bp	Bp	NT	-	+	-	+
B. pseudomallei	33	PHE	Bp	Bp	NT	-	+	-	+
B. pseudomallei	35	PHE	Bp	Bp	NT	-	+	-	+
B. pseudomallei	36	PHE	Bp	Bp	NT	-	+	-	+
B. pseudomallei	38	PHE	Bp	Bp	NT	-	+	-	+
B. pseudomallei	39	PHE	Bp	Bp	NT	-	+	-	+
B. pseudomallei	40	PHE	Bp	Bp	NT	-	+	-	+
B. pseudomallei	43	PHE	Bp	Bp	NT	-	+	-	+
B. pseudomallei	44	PHE	Bp	Bp	NT	-	+	-	+
B. pseudomallei	45	PHE	Bp	Bp	NT	-	+	-	+
B. pseudomallei	46	PHE	Bp	Bp	NT	-	+	-	+
B. pseudomallei	47	PHE	Bp	Bp	NT	-	+	-	+
B. pseudomallei	53	PHE	Bp	Bp	NT	-	+	-	+
B. pseudomallei	66	PHE	Bp	Bp	NT	-	+	-	+
B. pseudomallei	67	PHE	Bp	Bp	NT	-	+	-	+
B. pseudomallei	68	PHE	Bp	Bp	NT	-	+	-	+
B. pseudomallei	71	PHE	Bp	Bp	NT	-	+	-	+
B. pseudomallei	72	PHE	Bp	Bp	NT	-	+	-	+
B. pseudomallei	73	PHE	Bp	Bp	NT	-	+	-	+
B. pseudomallei	75	PHE	Bp	Bp	NT	-	+	-	+
B. pseudomallei	79	PHE	Bp	Bp	NT	-	+	-	+
B. pseudomallei	83	PHE	Bp	Bp	NT	-	+	-	+
B. pseudomallei	84	PHE	Bp	Bp	NT	-	+	-	+

**Species**	**Isolate**	**Source**	**GC-FAME (RBTR3)**	**U'Ren *et al.*, (2005) qPCR**	**Thibault *et al.*, (2004) qPCR**	**16.5 kDa (Bm)**	***Orf11* (Bp)**	**70 kDa (Bt)**	***fliC* (Bp Complex)**
B. pseudomallei	85	PHE	Bp	Bp	NT	-	+	-	+
B. pseudomallei	91	PHE	Bp	Bp	NT	-	+	-	+
B. pseudomallei	92	PHE	Bp	Bp	NT	-	+	-	+
B. pseudomallei	104	PHE	Bp	Bp	NT	-	+	-	+
B. pseudomallei	110	PHE	Bp	Bp	NT	-	+	-	+
B. pseudomallei	111	PHE	Bp	Bp	NT	-	+	-	+
B. pseudomallei	112	PHE	Bp	Bp	NT	-	+	-	+
B. pseudomallei	126	PHE	Bp	Bp	NT	-	+	-	+
B. pseudomallei	135*	PHE	Bm	-	-	-	-	-	-
B. pseudomallei	208	PHE	Bp	Bp	NT	-	+	-	+
B. pseudomallei	211	PHE	Bp	Bp	NT	-	+	-	+
B. pseudomallei	216	PHE	Bp	Bp	NT	-	+	-	+
B. pseudomallei	392	PHE	Bp	Bp	NT	-	+	-	+
B. pseudomallei	2889	PHE	Bp	Bp	NT	-	+	-	+
B. pseudomallei	3477	PHE	Bp	Bp	NT	-	+	-	+
B. pseudomallei	3584	PHE	Bp	Bp	NT	-	+	-	+
B. pseudomallei	3783	PHE	Bp	Bp	NT	-	+	-	+
B. pseudomallei	3811	PHE	Bp	Bp	NT	-	+	-	+
B. pseudomallei	3871	PHE	Bp	Bp	NT	-	+	-	+
B. pseudomallei	4045	PHE	Bp	Bp	NT	-	+	-	+
B. pseudomallei	4075	PHE	Bp	Bp	NT	-	+	-	+
B. pseudomallei	4151	PHE	Bp	Bp	NT	-	+	-	+
B. pseudomallei	4152	PHE	Bp	Bp	NT	-	+	-	+
B. pseudomallei	98/SID2953	PHE	Bp	Bp	NT	-	+	-	+
B. pseudomallei	98/SID3292	PHE	Bp	Bp	NT	-	+	-	+
B. pseudomallei	99/SID4349	PHE	Bp	Bp	NT	-	+	-	+
B. pseudomallei	8016	NCTC	Bp	Bp	NT	-	+	-	+
B. pseudomallei	10276	NCTC	Bp	Bp	NT	-	+	-	+
B. pseudomallei	11668	ATCC	Bp	Bp	NT	-	+	-	+
B. pseudomallei	13178	NCTC	Bp	Bp	NT	-	+	-	+
B. pseudomallei	15682	ATCC	Bp	Bp	NT	-	+	-	+

**Species**	**Isolate**	**Source**	**GC-FAME (RBTR3)**	**U'Ren *et al.*, (2005) qPCR**	**Thibault *et al.*, (2004) qPCR**	**16.5 kDa (Bm)**	***Orf11* (Bp)**	**70 kDa (Bt)**	***fliC* (Bp Complex)**
B. pseudomallei	23343	ATCC	Bp	Bp	NT	-	+	-	+
B. pseudomallei	G9313	CDC	Bp	Bp	NT	-	+	-	+
B. pseudomallei	G9709	CDC	Bp	Bp	NT	-	+	-	+
B. pseudomallei	H929	CDC	Bp	Bp	NT	-	+	-	+
B. pseudomallei	H1406B	CDC	Bp	Bp	NT	-	+	-	+
B. pseudomallei	H1442	CDC	Bp	Bp	NT	-	+	-	+
B. pseudomallei	H1689	CDC	Bp	Bp	NT	-	+	-	+
B. pseudomallei	K96243	NCTC	Bp	Bp	NT	-	+	-	+
B. pseudomallei	KC872	CDC	Bp	Bp	NT	-	+	-	+
B. pseudomallei	80800117	UDH	Bp	Bp	NT	-	+	-	+
B. pseudomallei	81300007	UDH	Bp	Bp	NT	-	+	-	+
B. pseudomallei	MSHR 126	RDH	Bp	Bp	NT	-	+	-	+
B. pseudomallei	MSHR 144	RDH	Bp	Bp	NT	-	+	-	+
B. pseudomallei	MSHR 145	RDH	Bp	Bp	NT	-	+	-	+
B. pseudomallei	MSHR 146	RDH	Bp	Bp	NT	-	+	-	+
B. pseudomallei	MSHR 157	RDH	Bp	Bp	NT	-	+	-	+
B. pseudomallei	MSHR 150	RDH	Bp	Bp	NT	-	+	-	+
B. pseudomallei	MSHR 151	RDH	Bp	Bp	NT	-	+	-	+
B. pseudomallei	MSHR 153	RDH	Bp	Bp	NT	-	+	-	+
B. pseudomallei	MSHR 155	RDH	Bp	Bp	NT	-	+	-	+
B. pseudomallei	MSHR 156	RDH	Bp	Bp	NT	-	+	-	+
B. pseudomallei	MSHR 186	RDH	Bp	Bp	NT	-	+	-	+
B. pseudomallei	MSHR 190	RDH	Bp	Bp	NT	-	+	-	+
B. pseudomallei	MSHR 191	RDH	Bp	Bp	NT	-	+	-	+
B. pseudomallei	MSHR 192	RDH	Bp	Bp	NT	-	+	-	+
B. pseudomallei	MSHR 193	RDH	Bp	Bp	NT	-	+	-	+
B. pseudomallei	MSHR 194	RDH	Bp	Bp	NT	-	+	-	+
B. pseudomallei	MSHR 197	RDH	Bp	Bp	NT	-	+	-	+
B. pseudomallei	MSHR 199	RDH	Bp	Bp	NT	-	+	-	+
B. pseudomallei	MSHR 200	RDH	Bp	Bp	NT	-	+	-	+
B. pseudomallei	MSHR 202	RDH	Bp	Bp	NT	-	+	-	+

Species	Isolate	Source	GC-FAME (RBTR3)	U'Ren *et al.*, (2005) qPCR	Thibault *et al.*, (2004) qPCR	16.5 kDa (Bm)	*Orf11* (Bp)	70 kDa (Bt)	*fliC* (Bp Complex)
B. pseudomallei	MSHR 203	RDH	Bp	Bp	NT	-	+	-	+
B. pseudomallei	MSHR 206	RDH	Bp	Bp	NT	-	+	-	+
B. pseudomallei	MSHR 207	RDH	Bp	Bp	NT	-	+	-	+
B. pseudomallei	MSHR 208	RDH	Bp	Bp	NT	-	+	-	+
B. pseudomallei	MSHR 209	RDH	Bp	Bp	NT	-	+	-	+
B. pseudomallei	MSHR 225	RDH	Bp	Bp	NT	-	+	-	+
B. pseudomallei	MSHR 255	RDH	Bp	Bp	NT	-	+	-	+
B. pseudomallei	MSHR 257	RDH	Bp	Bp	NT	-	+	-	+
B. pseudomallei	MSHR 267	RDH	Bp	Bp	NT	-	+	-	+
B. pseudomallei	MSHR 283	RDH	Bp	Bp	NT	-	+	-	+
B. pseudomallei	MSHR 284	RDH	Bp	Bp	NT	-	+	-	+
B. pseudomallei	MSHR 307	RDH	Bp	Bp	NT	-	+	-	+
B. pseudomallei	MSHR 309	RDH	Bp	Bp	NT	-	+	-	+
B. pseudomallei	MSHR 347	RDH	Bp	Bp	NT	-	+	-	+
B. pseudomallei	MSHR 351	RDH	Bp	Bp	NT	-	+	-	+
B. pseudomallei	MSHR 352	RDH	Bp	Bp	NT	-	+	-	+
B. pseudomallei	MSHR 357	RDH	Bp	Bp	NT	-	+	-	+
B. pseudomallei	MSHR 363	RDH	Bp	Bp	NT	-	+	-	+
B. pseudomallei	MSHR 368	RDH	Bp	Bp	NT	-	+	-	+
B. pseudomallei	MSHR 369	RDH	Bp	Bp	NT	-	+	-	+
B. pseudomallei	MSHR 370	RDH	Bp	Bp	NT	-	+	-	+
B. pseudomallei	MSHR 371	RDH	Bp	Bp	NT	-	+	-	+
B. pseudomallei	MSHR 395	RDH	Bp	Bp	NT	-	+	-	+
B. pseudomallei	MSHR 397	RDH	Bp	Bp	NT	-	+	-	+
B. pseudomallei	MSHR 398	RDH	Bp	Bp	NT	-	+	-	+
B. pseudomallei	MSHR 402	RDH	Bp	Bp	NT	-	+	-	+
B. pseudomallei	MSHR 403	RDH	Bp	Bp	NT	-	+	-	+
B. pseudomallei	MSHR 406	RDH	Bp	Bp	NT	-	+	-	+
B. pseudomallei	MSHR 414	RDH	Bp	Bp	NT	-	+	-	+
B. pseudomallei	MSHR 415	RDH	Bp	Bp	NT	-	+	-	+
B. pseudomallei	MSHR 417	RDH	Bp	Bp	NT	-	+	-	+

**Species**	**Isolate**	**Source**	**GC-FAME (RBTR3)**	**U'Ren *et al*., (2005) qPCR**	**Thibault *et al*., (2004) qPCR**	**16.5 kDa (Bm)**	***Orf11* (Bp)**	**70 kDa (Bt)**	***fliC* (Bp Complex)**
B. pseudomallei	MSHR 419	RDH	Bp	Bp	NT	-	+	-	+
B. pseudomallei	MSHR 434	RDH	Bp	Bp	NT	-	+	-	+
B. pseudomallei	MSHR 523	RDH	Bp	Bp	NT	-	+	-	+
B. pseudomallei	MSHR 630	RDH	Bp	Bp	NT	-	+	-	+
B. pseudomallei	MSHR 684	RDH	Bp	Bp	NT	-	+	-	+
B. pseudomallei	MSHR 695	RDH	Bp	Bp	NT	-	+	-	+
B. pseudomallei	MSHR 861	RDH	Bp	Bp	NT	-	+	-	+
B. pseudomallei	MSHR 867	RDH	Bp	Bp	NT	-	+	-	+
B. pseudomallei	MSHR 906	RDH	Bp	Bp	NT	-	+	-	+
B. pseudomallei	MSHR 1001	RDH	Bp	Bp	NT	-	+	-	+
B. pseudomallei	MSHR 1062	RDH	Bp	Bp	NT	-	+	-	+
B. pseudomallei	MSHR 1073	RDH	Bp	Bp	NT	-	+	-	+
B. pseudomallei	MSHR 1076	RDH	Bp	Bp	NT	-	+	-	+
B. pseudomallei	MSHR 1036	RDH	Bp	Bp	NT	-	+	-	+
B. pseudomallei	MSHR 1042	RDH	Bp	Bp	NT	-	+	-	+
B. pseudomallei	MSHR 1057	RDH	Bp	Bp	NT	-	+	-	+
B. pseudomallei	MSHR 1080	RDH	Bp	Bp	NT	-	+	-	+
B. pseudomallei	MSHR 1081	RDH	Bp	Bp	NT	-	+	-	+
B. pseudomallei	MSHR 1084	RDH	Bp	Bp	NT	-	+	-	+
B. pseudomallei	MSHR 1088	RDH	Bp	Bp	NT	-	+	-	+
B. pseudomallei	MSHR 1089	RDH	Bp	Bp	NT	-	+	-	+
B. pseudomallei	MSHR 1097	RDH	Bp	Bp	NT	-	+	-	+
B. pseudomallei	MSHR 1122	RDH	Bp	Bp	NT	-	+	-	+
B. pseudomallei	MSHR 1144	RDH	Bp	Bp	NT	-	+	-	+
B. pseudomallei	MSHR 1148	RDH	Bp	Bp	NT	-	+	-	+
B. pseudomallei	MSHR 1174	RDH	Bp	Bp	NT	-	+	-	+
B. pseudomallei	MSHR 1186	RDH	Bp	Bp	NT	-	+	-	+
B. pseudomallei	MSHR 1223	RDH	Bp	Bp	NT	-	+	-	+
B. pseudomallei	MSHR 1281	RDH	Bp	Bp	NT	-	+	-	+
B. pseudomallei	MSHR 1286	RDH	Bp	Bp	NT	-	+	-	+
B. pseudomallei	MSHR 1316	RDH	Bp	Bp	NT	-	+	-	+

**Species**	**Isolate**	**Source**	**GC-FAME (RBTR3)**	**U'Ren *et al.*, (2005) qPCR**	**Thibault *et al.*, (2004) qPCR**	**16.5 kDa (Bm)**	***Orf11* (Bp)**	**70 kDa (Bt)**	***fliC* (Bp Complex)**
B. pseudomallei	MSHR 1317	RDH	Bp	Bp	NT	-	+	-	+
B. pseudomallei	MSHR 1320	RDH	Bp	Bp	NT	-	+	-	+
B. pseudomallei	MSHR 1332	RDH	Bp	Bp	NT	-	+	-	+
B. pseudomallei	MSHR 1335	RDH	Bp	Bp	NT	-	+	-	+
B. pseudomallei	MSHR 1348	RDH	Bp	Bp	NT	-	+	-	+
B. pseudomallei	MSHR 1353	RDH	Bp	Bp	NT	-	+	-	+
B. pseudomallei	MSHR 1367	RDH	Bp	Bp	NT	-	+	-	+
B. pseudomallei	MSHR 1368	RDH	Bp	Bp	NT	-	+	-	+
B. pseudomallei	MSHR 1372	RDH	Bp	Bp	NT	-	+	-	+
B. pseudomallei	MSHR 1373	RDH	Bp	Bp	NT	-	+	-	+
B. pseudomallei	MSHR 1392	RDH	Bp	Bp	NT	-	+	-	+
B. pseudomallei	MSHR 1401	RDH	Bp	Bp	NT	-	+	-	+
B. pseudomallei	MSHR 1417	RDH	Bp	Bp	NT	-	+	-	+
B. pseudomallei	MSHR 1428	RDH	Bp	Bp	NT	-	+	-	+
B. pseudomallei	MSHR 1439	RDH	Bp	Bp	NT	-	+	-	+
B. pseudomallei	MSHR 1456	RDH	Bp	Bp	NT	-	+	-	+
B. pseudomallei	MSHR 1500	RDH	Bp	Bp	NT	-	+	-	+
B. pseudomallei	MSHR 1530	RDH	Bp	Bp	NT	-	+	-	+
B. pseudomallei	MSHR 1555	RDH	Bp	Bp	NT	-	+	-	+
B. pseudomallei	MSHR 1562	RDH	Bp	Bp	NT	-	+	-	+
B. pseudomallei	MSHR 1565	RDH	Bp	Bp	NT	-	+	-	+
B. pseudomallei	MSHR 1566	RDH	Bp	Bp	NT	-	+	-	+
B. pseudomallei	MSHR 1569	RDH	Bp	Bp	NT	-	+	-	+
B. pseudomallei	MSHR 1575	RDH	Bp	Bp	NT	-	+	-	+
B. pseudomallei	MSHR 1609	RDH	Bp	Bp	NT	-	+	-	+
B. pseudomallei	MSHR 1631	RDH	Bp	Bp	NT	-	+	-	+
B. pseudomallei	MSHR 1634	RDH	Bp	Bp	NT	-	+	-	+
B. pseudomallei	MSHR 1635	RDH	Bp	Bp	NT	-	+	-	+
B. pseudomallei	MSHR 1709	RDH	Bp	Bp	NT	-	+	-	+
B. pseudomallei	MSHR 1711	RDH	Bp	Bp	NT	-	+	-	+
B. pseudomallei	MSHR 1713	RDH	Bp	Bp	NT	-	+	-	+

Species	Isolate	Source	GC-FAME (RBTR3)	U'Ren *et al*., (2005) qPCR	Thibault *et al*., (2004) qPCR	16.5 kDa (Bm)	*Orf11* (Bp)	70 kDa (Bt)	*fliC* (Bp Complex)
B. pseudomallei	MSHR 1714	RDH	Bp	Bp	NT	-	+	-	+
B. pseudomallei	MSHR 1725	RDH	Bp	Bp	NT	-	+	-	+
B. pseudomallei	MSHR 1749	RDH	Bp	Bp	NT	-	+	-	+
B. pseudomallei	MSHR 1759	RDH	Bp	Bp	NT	-	+	-	+
B. pseudomallei	MSHR 1763	RDH	Bp	Bp	NT	-	+	-	+
B. pseudomallei	MSHR 1764	RDH	Bp	Bp	NT	-	+	-	+
B. pseudomallei	MSHR 1766	RDH	Bp	Bp	NT	-	+	-	+
B. pseudomallei	MSHR 1768	RDH	Bp	Bp	NT	-	+	-	+
B. pseudomallei	MSHR 1775	RDH	Bp	Bp	NT	-	+	-	+
B. pseudomallei	MSHR 1777	RDH	Bp	Bp	NT	-	+	-	+
B. pseudomallei	MSHR 1816	RDH	NMF	-	-	-	-	-	-
B. pseudomallei	MSHR 1829	RDH	Bp	Bp	NT	-	+	-	+
B. pseudomallei	MSHR 1840	RDH	Bp	Bp	NT	-	+	-	+
B. pseudomallei	MSHR 1848	RDH	Bp	Bp	NT	-	+	-	+
B. pseudomallei	MSHR 1859	RDH	Bp	Bp	NT	-	+	-	+
B. pseudomallei	MSHR 1865	RDH	Bp	Bp	NT	-	+	-	+
B. pseudomallei	MSHR 1866	RDH	Bp	Bp	NT	-	+	-	+
B. pseudomallei	MSHR 1868	RDH	Bp	Bp	NT	-	+	-	+
B. pseudomallei	MSHR 1884	RDH	Bp	Bp	NT	-	+	-	+
B. pseudomallei	MSHR 1886	RDH	Bp	Bp	NT	-	+	-	+
B. pseudomallei	MSHR 1890	RDH	Bp	Bp	NT	-	+	-	+
B. pseudomallei	MSHR 1891	RDH	Bp	Bp	NT	-	+	-	+
B. pseudomallei	MSHR 1893	RDH	Bp	Bp	NT	-	+	-	+
B. pseudomallei	MSHR 1895	RDH	Bp	Bp	NT	-	+	-	+
B. pseudomallei	MSHR 1896	RDH	Bp	Bp	NT	-	+	-	+
B. pseudomallei	MSHR 1905	RDH	Bp	Bp	NT	-	+	-	+
B. pseudomallei	MSHR 1912	RDH	Bm	Bp	NT	-	+	-	+
B. pseudomallei	MSHR 1917	RDH	Bp	Bp	NT	-	+	-	+
B. pseudomallei	MSHR 1926	RDH	Bp	Bp	NT	-	+	-	+
B. pseudomallei	MSHR 1946	RDH	Bp	Bp	NT	-	+	-	+
B. pseudomallei	MSHR 1953	RDH	Bp	Bp	NT	-	+	-	+

Species	Isolate	Source	GC-FAME (RBTR3)	U'Ren *et al.*, (2005) qPCR	Thibault *et al.*, (2004) qPCR	16.5 kDa (Bm)	*Orf11* (Bp)	70 kDa (Bt)	*fliC* (Bp Complex)
B. pseudomallei	MSHR 1954	RDH	Bm	Bp	NT	-	+	-	+
B. pseudomallei	MSHR 1967	RDH	Bp	Bp	NT	-	+	-	+
B. pseudomallei	MSHR 1986	RDH	Bm	Bp	NT	-	+	-	+
B. pseudomallei	MSHR 2002	RDH	Bp	Bp	NT	-	+	-	+
B. pseudomallei	MSHR 2003	RDH	Bp	Bp	NT	-	+	-	+
B. pseudomallei	MSHR 2012	RDH	Bp	Bp	NT	-	+	-	+
B. pseudomallei	MSHR 2024	RDH	Bp	Bp	NT	-	+	-	+
B. pseudomallei	MSHR 2030	RDH	Bp	Bp	NT	-	+	-	+
B. pseudomallei	MSHR 2037	RDH	Bp	Bp	NT	-	+	-	+
B. pseudomallei	MSHR 2040	RDH	Bp	Bp	NT	-	+	-	+
B. pseudomallei	MSHR 2052	RDH	Bp	Bp	NT	-	+	-	+
B. pseudomallei	MSHR 2053	RDH	Bp	Bp	NT	-	+	-	+
B. pseudomallei	MSHR 2080	RDH	Bp	Bp	NT	-	+	-	+
B. pseudomallei	MSHR 2084	RDH	Bp	Bp	NT	-	+	-	+
B. pseudomallei	MSHR 2085	RDH	Bp	Bp	NT	-	+	-	+
B. pseudomallei	MSHR 2086	RDH	Bp	Bp	NT	-	+	-	+
B. pseudomallei	MSHR 2158	RDH	Bp	Bp	NT	-	+	-	+
B. pseudomallei	MSHR 2168	RDH	Bp	Bp	NT	-	+	-	+
B. pseudomallei	MSHR 2170	RDH	Bp	Bp	NT	-	+	-	+
B. pseudomallei	MSHR 2174	RDH	Bp	Bp	NT	-	+	-	+
B. pseudomallei	MSHR 2201	RDH	Bp	Bp	NT	-	+	-	+
B. pseudomallei	MSHR 2208	RDH	Bp	Bp	NT	-	+	-	+
B. pseudomallei	MSHR 2212	RDH	Bp	Bp	NT	-	+	-	+
B. pseudomallei	MSHR 2213	RDH	Bp	Bp	NT	-	+	-	+
B. pseudomallei	MSHR 2214	RDH	Bp	Bp	NT	-	+	-	+
B. pseudomallei	MSHR 2221	RDH	Bp	Bp	NT	-	+	-	+
B. pseudomallei	MSHR 2226	RDH	Bp	Bp	NT	-	+	-	+
B. pseudomallei	MSHR 2227	RDH	Bp	Bp	NT	-	+	-	+
B. pseudomallei	MSHR 2232	RDH	Bp	Bp	NT	-	+	-	+
B. pseudomallei	MSHR 2235	RDH	Bp	Bp	NT	-	+	-	+
B. pseudomallei	MSHR 2236	RDH	Bp	Bp	NT	-	+	-	+

**Species**	**Isolate**	**Source**	**GC-FAME (RBTR3)**	**U'Ren *et al*., (2005) qPCR**	**Thibault *et al*., (2004) qPCR**	**16.5 kDa (Bm)**	***Orf11* (Bp)**	**70 kDa (Bt)**	***fliC* (Bp Complex)**
B. pseudomallei	MSHR 2238	RDH	Bp	Bp	NT	-	+	-	+
B. pseudomallei	MSHR 2243	RDH	Bp	Bp	NT	-	+	-	+
B. pseudomallei	MSHR 2245	RDH	Bp	Bp	NT	-	+	-	+
B. pseudomallei	MSHR 2251	RDH	Bp	Bp	NT	-	+	-	+
B. pseudomallei	MSHR 2255	RDH	Bp	Bp	NT	-	+	-	+
B. pseudomallei	MSHR 2258	RDH	Bp	Bp	NT	-	+	-	+
B. pseudomallei	MSHR 2263	RDH	Bp	Bp	NT	-	+	-	+
B. pseudomallei	MSHR 2269	RDH	Bp	Bp	NT	-	+	-	+
B. pseudomallei	MSHR 2350	RDH	Bp	Bp	NT	-	+	-	+
B. pseudomallei	MSHR 2351	RDH	Bp	Bp	NT	-	+	-	+
B. pseudomallei	MSHR 2366	RDH	Bp	Bp	NT	-	+	-	+
B. pseudomallei	MSHR 2375	RDH	Bp	Bp	NT	-	+	-	+
B. pseudomallei	MSHR 2380	RDH	Bp	Bp	NT	-	+	-	+
B. pseudomallei	MSHR 2381	RDH	Bp	Bp	NT	-	+	-	+
B. pseudomallei	MSHR 2385	RDH	Bp	Bp	NT	-	+	-	+
B. pseudomallei	MSHR 2386	RDH	Bp	Bp	NT	-	+	-	+
B. pseudomallei	MSHR 2388	RDH	Bp	Bp	NT	-	+	-	+
B. pseudomallei	MSHR 2391	RDH	Bp	Bp	NT	-	+	-	+
B. pseudomallei	MSHR 2393	RDH	Bp	Bp	NT	-	+	-	+
B. pseudomallei	MSHR 2394	RDH	Bc/Bpy/Ba	Bp	NT	-	+	-	+
B. pseudomallei	MSHR 2395	RDH	Bp	Bp	NT	-	+	-	+
B. pseudomallei	MSHR 2398	RDH	Bp	Bp	NT	-	+	-	+
B. pseudomallei	MSHR 2400	RDH	Bp	Bp	NT	-	+	-	+
B. pseudomallei	MSHR 2403	RDH	Bp	Bp	NT	-	+	-	+
B. pseudomallei	MSHR 2409	RDH	Bp	Bp	NT	-	+	-	+
B. ambifaria	BAA-244	ATCC	NT	-	-	-	-	-	-
B. andropogonis	23061	ATCC	NT	-	-	-	-	-	-
B. cepacia	10856	ATCC	NT	-	-	-	-	-	-
B. cepacia	25416	ATCC	NT	-	-	-	-	-	-
B. gladioli	10248	ATCC	NT	-	-	-	-	-	-

Species	Isolate	Source	GC-FAME (RBTR3)	U'Ren *et al*., (2005) qPCR	Thibault *et al*., (2004) qPCR	16.5 kDa (Bm)	*Orf11* (Bp)	70 kDa (Bt)	*fliC* (Bp Complex)
B. glathei	29196	ATCC	NT	-	-	-	-	-	-
B. graminis	700544	ATCC	NT	-	-	-	-	-	-
B. multivorans	BAA-247	ATCC	NT	-	-	-	-	-	-
B. pyrrocina	15958	ATCC	NT	-	-	-	-	-	-
B. sordidicola	49583	CCUG	NT	-	-	-	-	-	-
B. vandii	51545	ATCC	NT	-	-	-	-	-	-
B. vietnamiensis	BAA-248	ATCC	NT	-	-	-	-	-	-
P. aeruginosa	15442	ATCC	NT	-	-	-	-	-	-
R. solancearum	11696	ATCC	NT	-	-	-	-	-	-
S. maltophilia	13637	ATCC	NT	-	-	-	-	-	-

Ba, *B. ambifaria*; Bc, *B. cenocepacia*; Bm, *B. mallei*; Bp, *B. pseudomallei*; Bpy, *B. pyrrocinia*; NMF, No Matches Found; NT, Not tested; *, Misclassified bacterial isolates

ATCC, American Type Culture Collection; CCUG, Culture Collection, University of Göteborg; CDC, Centers for Disease Control and Prevention; RDH, Royal Darwin Hospital; NCTC, National Collection of Type Cultures; PHE, Public Health England; UC, University of Calgary; UDH, Utah Department of Health
