# Supplementary material for: A Quadruplex Real-Time PCR Assay for the Rapid Detection and Differentiation of the Most Relevant Members of the B. pseudomallei Complex: B. mallei, B. pseudomallei, and B. thailandensis
Source: PLoS One. 2016 Oct 13;11(10):e0164006. doi: 10.1371/journal.pone.0164006 (PMC5063335; doi:10.1371/journal.pone.0164006)
Supplement: S2 Table — (PDF) [file pone.0164006.s005.pdf]

Species	Country of Origin	Source	No. of Isolates
B. mallei	China	Animal	1
	China	Human	1
	Hungary	Animal	1
	Hungary	Unknown	1
	India	Animal	2
	India	Unknown	1
	Turkey	Human	2
	Turkey	Unknown	1
	United Kingdom	Unknown	1
	Unknown	Animal	1
	Unknown	Unknown	1
	**5 (Total)**		**13 (Total)**
B. thailandensis	Thailand	Environmental	10
	France	Animal	1
	**2 (Total)**	1	**11 (Total)**
B. pseudomallei	Australia	Animal	54
	Australia	Environmental	32
	Australia	Human	121*
	Bangladesh	Human	1
	Ecuador	Human	1
	Fiji	Human	1
	France	Animal	1
	France	Environmental	1
	Holland	Human	3
	Indonesia	Animal	3
	Italy	Human	1
	Kenya	Environmental	1
	Kenya	Human	1
	Madagascar	Environmental	1
	Malaysia	Human	5
	Pakistan	Human	1
	Papua New Guinea	Human	1
	Philippines	Animal	2
	Singapore	Environmental	1
	Singapore	Human	4
	Sweden	Human	1
	Thailand	Environmental	1
	Thailand	Human	13
	Thailand	Environmental	1*
	United Kingdom	Human	8
	United Kingdom	Unknown	1
	United States	Human	2
	Venezuela	Unknown	1
	Vietnam	Human	1
	Unknown	Animal	1
	Unknown	Human	1
	Unknown	Unknown	8
	**21 (Total)**		**275 (Total)**

*****An isolate from this category is misclassified
