# Supplementary material for: A Quadruplex Real-Time PCR Assay for the Rapid Detection and Differentiation of the Most Relevant Members of the B. pseudomallei Complex: B. mallei, B. pseudomallei, and B. thailandensis
Source: PLoS One. 2016 Oct 13;11(10):e0164006. doi: 10.1371/journal.pone.0164006 (PMC5063335; doi:10.1371/journal.pone.0164006)
Supplement: S3 Table — The multiplex assay was evaluated against all completed sequences of B. mallei, B. pseudomallei, B. thailandensis, B. oklahomensis, B. humptydooensis strains along with a few other Burkholderia near-neighbors from the NCBI GenBank database. (PDF) [file pone.0164006.s006.pdf]

**Species**	**Isolate**	**Accession Number**	**16.5 kDa (Bm)**	***Orf11* (Bp)**	**70 kDa (Bt)**	***fliC* (Bp, Bm, Bt, Bo Complex)**
B. mallei	6	CP008711	+	-	-	+
B. mallei	11	CP009587	+	-	-	+
B. mallei	23344	CP008704	+	-	-	+
B. mallei	2000031063	CP008732	+	-	-	+
B. mallei	2002721276	CP010065	+	-	-	+
B. mallei	2002734299	CP009337	+	-	-	+
B. mallei	2002734306	CP009707	+	-	-	+
B. mallei	BMQ	CP008723	+	-	-	+
B. mallei	India86-567-2	CP009642	+	-	-	+
B. mallei	FMH 23344	CP009148	+	-	-	+
B. mallei	KC 1092	CP009942	+	-	-	+
B. mallei	NCTC 10229	CP000546	+	-	-	+
B. mallei	NCTC 10247	CP007802	+	-	-	+
B. mallei	SAVP1	CP000526	+	-	-	+
B. thailandensis	34	CP010017	-	-	+	++
B. thailandensis	2002721643	CP009601	-	-	+	+
B. thailandensis	2002721723	CP004097	-	-	+	+
B. thailandensis	2003015869	CP008914	-	-	+	++
B. thailandensis	E254	CP004381	-	-	+	+
B. thailandensis	E264	CP008786	-	-	+	+
B. thailandensis	E444	CP004117	-	-	+	+
B. thailandensis	H0587	CP004089	-	-	+	++
B. thailandensis	MSMB 59	CP004385	-	-	+	+
B. thailandensis	USAMRU Malaysia #20	CP004383	-	-	+	+
B. pseudomallei	9	CP008755	-	+	-	+
B. pseudomallei	406e	CP009298	-	+	-	+
B. pseudomallei	576	CP008777	-	+	-	+
B. pseudomallei	982	CP012576	-	+	-	+
B. pseudomallei	1026b	CP004379	-	+	-	+
B. pseudomallei	1106a	CP008758	-	+	-	+
B. pseudomallei	1710b	CP000124	-	+	-	+
B. pseudomallei	7894	CP009535	-	+	-	+
B. pseudomallei	13179	CP003977	-	+	-	+
B. pseudomallei	350105	CP012094	-	+	-	+
B. pseudomallei	A79A	CP009165	-	+	-	+
B. pseudomallei	B03	CP009151	-	+	-	+
B. pseudomallei	BDP	CP009209	-	++	-	+
B. pseudomallei	BGK	CP008916	-	+	-	+
B. pseudomallei	BGR	CP008834	-	+	-	+

Species	Isolate	Accession Number	16.5 kDa (Bm)	*Orf11* (Bp)	70 kDa (Bt)	*fliC* (Bp, Bm, Bt, Bo Complex)
B. pseudomallei	Bp1651	CP012041	-	++	-	+
B. pseudomallei	BP 3921g	LK936442	-	+	-	+
B. pseudomallei	BPC006	CP003781	-	+	-	+
B. pseudomallei	BSR	CP009128	-	+	-	+
B. pseudomallei	HBPUB10134a	CP008911	-	++	-	+
B. pseudomallei	HBPUB10303a	CP008894	-	+	-	+
B. pseudomallei	K42	CP009162	-	++	-	+
B. pseudomallei	K96243	CP009538	-	+	-	+
B. pseudomallei	Mahidol-1106a	CP008781	-	+	-	+
B. pseudomallei	MSHR 62	CP009235	-	+	-	+
B. pseudomallei	MSHR 146	CP004042	-	++	-	+
B. pseudomallei	MSHR 305	CP006470	-	++	-	+
B. pseudomallei	MSHR 346	CP008764	-	++	-	+
B. pseudomallei	MSHR 491	CP009485	-	++	-	+
B. pseudomallei	MSHR 511	CP004023	-	++	-	+
B. pseudomallei	MSHR 520	CP004368	-	++	-	+
B. pseudomallei	MSHR 668	CP009545	-	++	-	+
B. pseudomallei	MSHR 840	CP009474	-	++	-	+
B. pseudomallei	MSHR 1153	CP009271	-	+	-	+
B. pseudomallei	MSHR 1655	CP008780	-	+	-	++
B. pseudomallei	MSHR 2243	CP009270	-	+	-	+
B. pseudomallei	MSHR 2543	CP009478	-	+	-	+
B. pseudomallei	MSHR 3965	CP009153	-	+	-	+
B. pseudomallei	MSHR 5848	CP008909	-	+	-	+
B. pseudomallei	MSHR 5855	CP008784	-	+	-	+
B. pseudomallei	MSHR 5858	CP008892	-	+	-	+
B. pseudomallei	NAU20B-16	CP004003	-	++	-	+
B. pseudomallei	NAU35A-3	CP004377	-	+	-	+
B. pseudomallei	NCTC 13178	CP004001	-	+	-	+
B. pseudomallei	Pasteur 52237	CP009899	-	+	-	+
B. pseudomallei	PB08298010	CP009551	-	++	-	+
B. pseudomallei	PHLS 112	CP009585	-	++	-	+
B. pseudomallei	TSV48	CP009161	-	++	-	+
B. pseudomallei	TSV202	CP009157	-	+	-	++
B. pseudomallei	vgh07	CP010973	-	+	-	+
B. pseudomallei	vgh16R	CP012515	-	+	-	+
B. pseudomallei	vgh16W	CP012517	-	+	-	+
B. oklahomensis	C6786	CP009555	-	-	-	++
B. oklahomensis	EO147	CP008726	-	-	-	++
B. cenocepacia	MC0-3	CP000959	-	-	-	-
B. cepacia	GG4	CP003775	-	-	-	-

Species	Isolate	Accession Number	16.5 kDa (Bm)	*Orf11* (Bp)	70kDa (Bt)	*fliC* (Bp, Bm, Bt, Bo Complex)
B. dolosa	AU0158	CP009794	-	-	-	-
B. humptydooensis	MSMB 43	CP013382	-	-	-	-
B. humptydooensis	MSMB 121	CP004096	-	-	-	-
B. multivorans	ATCC 17616	AP009386	-	-	-	-
B. ubonensis	MSMB22	CP009486	-	-	-	-
B. vietnamiensis	LMG 10929	CP009631	-	-	-	-

+, indicates the assays will detect the indicated *Burkholderia* strain containing an exact match

++, indicates the assay will detect the indicated *Burkholderia* strain containing a mismatch

**Isolate**	***orf11* Forward T-G Mismatch 1** FoD: 0.991	***orf11* Forward G-G Mismatch 2** FoD: 0.926	***orf11* Reverse A-C Mismatch** FoD: 0.989	***orf11* Probe G-T Mismatch** FoD: 0.992
Mismatch profile 1				
Bp HBPUB10134a	x		x	
Bp PHLS 112*	x		x	
Mismatch profile 2				
Bp BDP		x	x	
Bp MSHR 305		x	x	
Bp MSHR 520		x	x	
Mismatch profile 3				
Bp Bp1651			x	
Bp K42			x	
Bp MSHR146*			x	
Bp MSHR 346			x	
Bp MSHR 511			x	
Bp PB08298010			x	
Bp NAU20B-16			x	
Bp TSV48			x	
			x	
Mismatch profile 4				
Bp MSHR 391			x	x
Bp MSHR 491			x	x
Bp MSHR 668			x	x

FoD, Fraction of duplex; Bp, *B. pseudomallei*; Bt, *B. thailandensis*; *, indicates sample was previously tested by the *Bcom* assay

**Isolate**	***fliC* Probe G-T Mismatch 1**<br>FoD: 0.997	***fliC* Probe C-C Mismatch 2**<br>FoD: 0.985	***fliC* Forward C-C Mismatch**<br>FoD: 0.940
Mismatch profile 1			
Bp TSV202	x		
Bp MSHR 1655	x		
Mismatch profile 2			
Bt 34		x	
Bt 2003015869		x	
Bt H0587		x	
Mismatch profile 3			
Bo C6786		x	x
Bo EO147		x	x

FoD, Fraction of duplex; Bp, *B. pseudomallei*; Bt, *B. thailandensis*; Bo, *B. oklahomensis*
