# Supplementary material for: A Quadruplex Real-Time PCR Assay for the Rapid Detection and Differentiation of the Most Relevant Members of the B. pseudomallei Complex: B. mallei, B. pseudomallei, and B. thailandensis
Source: PLoS One. 2016 Oct 13;11(10):e0164006. doi: 10.1371/journal.pone.0164006 (PMC5063335; doi:10.1371/journal.pone.0164006)
Supplement: S4 Table — Fraction of duplex values were calculated using DNA Thermodynamic & Hybridization software from Integrated DNA Technologies to determine the binding efficiency of an oligo when single nucleotide polymorphisms are present. A FoD value closer to 1 indicates a higher likelihood of proper binding. (PDF) [file pone.0164006.s007.pdf]

**Isolate**	***orf11* Forward T-G Mismatch 1** FoD: 0.991	***orf11* Forward G-G Mismatch 2** FoD: 0.926	***orf11* Reverse A-C Mismatch** FoD: 0.989	***orf11* Probe G-T Mismatch** FoD: 0.992
Mismatch profile 1				
Bp HBPUB10134a	x		x	
Bp PHLS 112*	x		x	
Mismatch profile 2				
Bp BDP		x	x	
Bp MSHR 305		x	x	
Bp MSHR 520		x	x	
Mismatch profile 3				
Bp Bp1651			x	
Bp K42			x	
Bp MSHR146*			x	
Bp MSHR 346			x	
Bp MSHR 511			x	
Bp PB08298010			x	
Bp NAU20B-16			x	
Bp TSV48			x	
			x	
Mismatch profile 4				
Bp MSHR 391			x	x
Bp MSHR 491			x	x
Bp MSHR 668			x	x

FoD, Fraction of duplex; Bp, *B. pseudomallei*; Bt, *B. thailandensis*; *, indicates sample was previously tested by the *Bcom* assay
