# Supplementary material for: A Quadruplex Real-Time PCR Assay for the Rapid Detection and Differentiation of the Most Relevant Members of the B. pseudomallei Complex: B. mallei, B. pseudomallei, and B. thailandensis
Source: PLoS One. 2016 Oct 13;11(10):e0164006. doi: 10.1371/journal.pone.0164006 (PMC5063335; doi:10.1371/journal.pone.0164006)
Supplement: S5 Table — Fraction of duplex values were calculated using DNA Thermodynamics & Hybridization software from Integrated DNA Technologies to determine the binding efficiency of an oligo when single nucleotide polymorphisms are present. A FoD value closer to 1 indicates a higher likelihood of proper binding. The software is unable to calculate degenerate bases; therefore, both bases were calculated and the average FoD is presented in this table. (PDF) [file pone.0164006.s008.pdf]

**Isolate**	***fliC* Probe G-T Mismatch 1** FoD: 0.997	***fliC* Probe C-C Mismatch 2** FoD: 0.985	***fliC* Forward C-C Mismatch** FoD: 0.940
Mismatch profile 1			
Bp TSV202	x		
Bp MSHR 1655	x		
Mismatch profile 2			
Bt 34		x	
Bt 2003015869		x	
Bt H0587		x	
Mismatch profile 3			
Bo C6786		x	x
Bo EO147		x	x

FoD, Fraction of duplex; Bp, *B. pseudomallei*; Bt, *B. thailandensis*; Bo, *B. oklahomensis*
